# ‘Clinically unnecessary’ use of emergency and urgent care: A realist review of patients' decision making

**DOI:** 10.1111/hex.12995

**Published:** 2019-10-29

**Authors:** Alicia O'Cathain, Janice Connell, Jaqui Long, Joanne Coster

**Affiliations:** ^1^ School of Health and Related Research (ScHARR) University of Sheffield Sheffield UK

**Keywords:** emergency medicine, heath care seeking behaviour, patients, urgent care

## Abstract

**Background:**

Demand is labelled ‘clinically unnecessary’ when patients do not need the levels of clinical care or urgency provided by the service they contact.

**Objective:**

To identify programme theories which seek to explain why patients make use of emergency and urgent care that is subsequently judged as clinically unnecessary.

**Design:**

Realist review.

**Methods:**

Papers from four recent systematic reviews of demand for emergency and urgent care, and an updated search to January 2017. Programme theories developed using Context‐Mechanism‐Outcome chains identified from 32 qualitative studies and tested by exploring their relationship with existing health behaviour theories and 29 quantitative studies.

**Results:**

Six mechanisms, based on ten interrelated programme theories, explained why patients made clinically unnecessary use of emergency and urgent care: (a) need for risk minimization, for example heightened anxiety due to previous experiences of traumatic events; (b) need for speed, for example caused by need to function normally to attend to responsibilities; (c) need for low treatment‐seeking burden, caused by inability to cope due to complex or stressful lives; (d) compliance, because family or health services had advised such action; (e) consumer satisfaction, because emergency departments were perceived to offer the desired tests and expertise when contrasted with primary care; and (f) frustration, where patients had attempted and failed to obtain a general practitioner appointment in the desired timeframe. Multiple mechanisms could operate for an individual.

**Conclusions:**

Rather than only focusing on individuals' behaviour, interventions could include changes to health service configuration and accessibility, and societal changes to increase coping ability.

## 1 BACKGROUND

When people want health advice or treatment urgently, they seek it from a number of health services including emergency ambulance services, emergency departments, general practice out of hours services, daytime general practice, urgent care centres, walk‐in centres, minor injury units, dentists and 24 hour telephone health helplines.^1^ The options available vary considerably between and within different countries. Concern has been expressed about high levels of demand for some of these services, specifically emergency ambulances, emergency departments and general practice.^2^, ^3^ These concerns sometimes focus on demand from patients who do not need the clinical resources or level of urgency of those services. These patients have been described variously as contacting emergency or urgent care services with minor, non‐urgent, non‐serious, medically unnecessary or low acuity problems,^4^, ^5^, ^6^ or more pejoratively as ‘inappropriate users’^7^ In this article, we use the term ‘clinically unnecessary’ in recognition that health professionals view some users as not requiring the level of clinical care provided by their service and who could be treated effectively by a lower urgency service.

Understanding why patients make decisions that are judged clinically unnecessary is important because this may inform interventions to reduce demand for overloaded health services. However, it is also important to be aware that patient behaviour is only one part of the picture. The concept of clinically unnecessary use of health services is contentious.^8^, ^9^ Patients face a moral dilemma in help‐seeking,^10^ anxious to take responsibility for their health whilst not being judged as wasting the time of a busy service.^11^ Judgements about the clinical necessity of demand may be shaped by the supply of services,^8^ where these judgements become harsher as demand outstrips supply. Staff judgements regarding legitimate reasons for service use may also vary between individual clinicians and individual services.^12^

Existing evidence provides some insights into this complex issue. A recent rapid review of qualitative, quantitative and mixed methods studies primarily from the United States and the United Kingdom^13^ identified six reasons for attendance at emergency and urgent care services: a lack of access to and confidence in primary care; perceptions of urgency or anxiety creating a need for reassurance from emergency services; recommendations to attend from friends or family or health‐care professionals; convenience in terms of services having better opening hours or being located closer to home than alternatives; patient factors such as lower cost than other options or lack of transport; and perceived need for treatment and investigations available at the hospital. Another recent systematic review, focusing more narrowly on reasons for self‐referral to emergency departments, identified a similar set of issues.^14^ A systematic review of use of ambulance services for primary care‐sensitive conditions included the perspectives of health professionals and service managers as well as patients.^15^ This found a somewhat different set of factors, albeit with some overlap with Coster et al^13^: poor physical health including comorbidities and mental health; personal anxiety and risk management; health knowledge; care givers and bystanders encouraging use of ambulances particularly for children; socio‐demographic and economic issues including deprivation and having no own transport; and poor access to primary care.

Whilst these systematic reviews provide valuable high‐quality evidence related to this issue, there is a need for a more in‐depth understanding of what drives patients to seek care urgently when it is clinically unnecessary. Existing reviews have focused on one service only,^14^, ^15^ included health professional as well as patient perspectives,^15^ or addressed overall demand, including both clinically necessary and unnecessary use.^13^ Therefore, there is a need to undertake an in‐depth review that focuses specifically on patients' perspectives of clinically unnecessary service use, to understand more about what drives them to seek care urgently, and attempts to gain a deeper understanding about the reasons for their decisions. Realist synthesis, which focuses on mechanisms that cause outcomes, and the contexts that shape these mechanisms and outcomes, could complement recent reviews by offering a more in‐depth understanding of patients' decision‐making processes. The aim of this review was therefore to use realist synthesis to identify patients' perspectives of why they make use of services providing emergency and urgent care that is judged clinically unnecessary.

## 2 METHODS

### 2.1 Realist synthesis

Realist synthesis is used to understand complex social programmes that involve human decisions and actions.^16^ Whilst it is usually used to explore how the outcomes of programmes or interventions are achieved, it has provided valuable insights outside the context of intervention research, including understanding access to primary care for socioeconomically disadvantaged older people in rural areas.^17^ Due to the complexity of decision making, and our desire to understand the mechanisms driving clinically unnecessary use, we considered realist synthesis to be an appropriate approach for this study.

We identified our outcome of interest as the use of an emergency and urgent care service that was judged as clinically unnecessary. We then undertook the review in two phases. The first phase involved developing and refining a set of programme theories based on qualitative research. The second phase involved testing these programme theories using existing theories of health behaviour and identifying evidence to support or refute them in relevant quantitative studies. We registered the proposal with PROSPERO 2017:CRD42017056273. We used the RAMESES reporting guidelines.^16^

### 2.2 Phase 1: Developing and refining the programme theories

#### 2.2.1 Initial theoretical framework

In realist synthesis, the initial theoretical framework or rough programme theories can be identified in different ways.^18^ The research team can draw on a combination of existing theories, published evidence and expert opinion.^18^ We used published evidence from a recently completed rapid review of demand for emergency and urgent care^13^ which offered a set of potential rough programme theories based on qualitative and quantitative research of all users of a range of emergency urgent care services. Because Coster et al's review^13^ did not focus solely on patients judged to have made clinically unnecessary use of services, we used this review as an overarching theoretical framework rather than a source of rough programme theories.

#### 2.2.2 Identification and selection of studies for inclusion

Realist synthesis does not necessarily limit itself to including only one study design but is adaptable to the particular context of the research.^16^ In this instance, we developed our programme theories only using journal articles reporting qualitative research or qualitative components of mixed methods studies because these offered insights based on in‐depth exploration of patient perspectives. Because a number of substantive reviews had already been published^13^, ^14^, ^15^ or were ongoing (Turnbull et al https://www.southampton.ac.uk/healthsciences/research/projects/a-study-of-sense-making-strategies-and-help-seeking-behaviours.page), we searched for relevant papers included in these four reviews.

**Figure 1:**
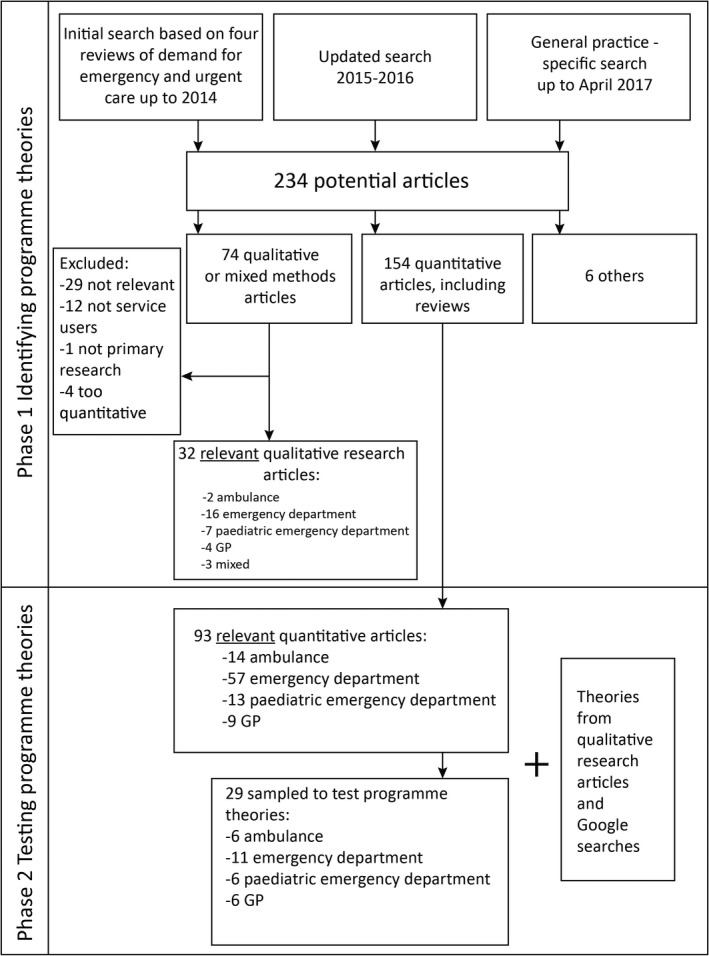
Summary of search, selection and extraction of articles

Coster et al^13^ had searched MEDLINE, Embase, Cochrane Library, Web of Science and CINAHL 1995‐2016. Kraaijvanger et al^14^ had searched MEDLINE, Embase, Cochrane Library, CINAHL and PubMed up to February 2015. Booker et al^15^ had searched MEDLINE, Embase, PsycINFO, Web of Science and CINAHL 1980 to June 2014. Turnbull et al (ongoing at the time of our review) had searched policy and published research MEDLINE, Embase, Web of Science, CINAHL and PsycINFO 1990 to 2017; their search only included articles up to 2016 at the time they shared their database with us in February 2017. To bring this evidence up to date, in February 2017 we undertook searches of MEDLINE and Google Scholar for any further articles published between 2015 and 2016. Due to the lack of articles focusing on clinically unnecessary use of daytime general practice within the four reviews, in April 2017 we searched MEDLINE and Google Scholar for relevant general practice focused studies from the start of the databases to March 2017. Figure 1 provides a summary of searches and the selection of studies. All included articles were written in English because this had been an inclusion criterion for the four reviews and the updated searches. Research from any country was included.

#### 2.2.3 Quality appraisal and data extraction

Realist synthesis does not employ the formal quality assessment process undertaken within other evidence synthesis approaches.^16^ The primary concern is the relevance of the material to the research question. Two researchers (JCon, JCos) screened each article reporting qualitative research for relevance in terms of its degree of focus on clinically unnecessary demand and its focus on patient perspectives. Where the explicit focus was on patients who were described as low triage category, low acuity, or using emergency care for an urgent, non‐urgent or primary care problem, we graded the article as 1 = directly relevant. Where the authors focused on a specific population sub‐group with the implication that they tend to make more clinically unnecessary use of services, we graded the article 2 = partially relevant. Articles focusing on frequent users of emergency departments were graded 2 because we felt that this group was a highly specific group within clinically unnecessary use and needed to be treated with care within the review. Articles exploring general demand for, or perceptions of, emergency and urgent care were graded 3 = not relevant and excluded. A third researcher (JL) checked the grading of each article identified as 1 or 2. Data extraction was undertaken by JCon and JL to produce a table documenting author, year, country, emergency/urgent care service, aim, data collection method, number and type of participants and key themes (Appendix S1). JL applied CASP quality criteria to included articles to consider the rigour of the included articles. We did not exclude articles based on rigour but instead identified articles where there were concerns about rigour and ensured that our programme theories did not rely solely on such articles as we developed and refined them.

#### 2.2.4 Developing and refining programme theories

JCon, JCos and AOC read a small number of the qualitative research articles to identify context (C) and mechanism (M) chains for the outcome (O) of using a higher acuity service than necessary. We undertook duplicate data extraction on these articles and discussed CMO chains and potential programme theories. The mechanism was defined as the trigger or driver for the decision, arising from an ongoing contextual situation, and the outcome as the service they chose to contact. The articles considered decision making both in relation to whether to seek help from a service urgently and which service to then contact. It was often difficult to distinguish context and mechanism,^19^, ^20^ because there were multiple mechanisms, some of which were often contexts for further mechanisms.

After learning from this exercise, formal analysis started with articles on emergency departments before moving on to ambulance services, general practice and finally multiple services. In July 2017, JCon presented the initial CMO chains based on 14 emergency department articles to our wider project team for discussion. This project team consisted of researchers in emergency and urgent care, three public and patient involvement representatives, a general practitioner and an emergency department consultant. After this presentation JCon continued to develop and refine the programme theories based on the remaining articles. For each service, the focus was on articles rated relevance = 1 before moving on to those rated relevance = 2. JCon, JL and AOC continued to discuss the CMO chains until we finalized 10 detailed programme theories. We presented the programme theories at a health services research conference (July 2018) and to our wider project team which included four public and patient representatives (October 2018). The wider project team challenged us to be clearer about the specific mechanisms driving the need for urgency, and this led to further discussion through which we identified 6 underlying mechanisms within the 10 programme theories. Finally, we presented these 6 underlying mechanisms and 10 programme theories for discussion to the project advisory group where members had backgrounds in emergency department medicine, paramedic practice, health‐care commissioning, research in emergency and urgent care, policy making and patient and public involvement (October 2018).

### 2.3 Phase 2: Testing the programme theories

Testing programme theories where an intervention is not the focus of the review is challenging. We chose to test the programme theories in two ways. First, through testing their relationship with existing theory about health behaviour because these encompass in‐depth understanding of the wider area of health behaviour. Second, by seeing if the programme theories had been identified in quantitative studies and if patients identified as making clinically unnecessary use of services were more likely to exhibit aspects of these programme theories.

In September 2017, whilst the programme theories were under development and refinement, JL and AOC used two approaches to search for existing theories relating to the evolving programme theories. Where included qualitative articles made reference to relevant theoretical work (perceptions of risk, coping under stress, perceptions of service provision), these references were followed up by JL, who then identified further literature relating to these theories or models, including any research specific to clinically unnecessary use of emergency and urgent care. Where there were no or a few references within the included articles that related to an evolving programme theory (fear or anxiety, uncertainty, influence of family and friends), AOC and JL undertook Google searches to identify relevant theoretical literature, particularly anything focusing on clinically unnecessary use of emergency and urgent care. These searches identified a key article integrating three existing theories of how people respond to symptoms to understand help‐seeking and illness behaviour.^21^

In September 2018, AOC returned to the 154 quantitative articles identified in the original searches (see Figure 1), the four articles excluded from Phase 1 because they were too quantitative (eg they reported qualitative research using percentages), and an extra relevant review identified in Google searches that had not been included in Phase 1. AOC screened these articles for relevance, that is identifying those focusing on clinically unnecessary use of services. AOC then undertook purposive sampling of different health services: ambulance, emergency department, paediatric emergency department/emergency department used for children, and general practice/mixed services. AOC ordered the articles about ambulance services by whether they were reviews or primary research and then by year of publication. AOC sampled recent reviews if these existed, and the most recent primary research articles. AOC repeated this process for articles about emergency departments, paediatric emergency departments and general practice. Because of the large number of articles on emergency departments, some sampling was also undertaken to include those with any emphasis on theory. There were only 3 relevant articles related to general practice so articles that were not directly relevant were included to offer further insights into this service. AOC extracted descriptive information for the 29 included articles (Appendix S1) and evidence supporting or refuting the 10 programme theories. The evidence consisted of cross‐sectional surveys of service users labelled as clinically unnecessary or comparisons of clinically unnecessary users with clinically necessary users.

### 2.4 Changes to original proposal

We made two changes to the original proposal. First, we did not undertake an appraisal of methodological rigour of all articles as planned. Not all realist reviews undertake methodological rigour. We focused on the rigour of the qualitative research used to develop and refine the programme theories because we wanted to ensure these were based on high‐quality research. Methodological rigour is not relevant to existing theory so we did not attempt to apply criteria to existing theories. Second, originally we planned to select 3‐6 rough programme theories to follow up but our evolving programme theories were interrelated and we considered them to be equally important and so followed up all 10 identified.

## 3 RESULTS

### 3.1 Description of the qualitative evidence base

32 articles reporting qualitative research were included: 18 were rated 1 ‘directly relevant’ and 14 were rated 2 ‘partially relevant’. The articles largely focused on emergency departments, either adult/mixed (16) or paediatric (7). Only two studies focused on ambulance services and four on general practitioner (GP) out of hours services. There were none from day time general practice. Articles were mainly from USA (12) and the UK (10), with others from continental Europe (5), Australia, Canada and the Caribbean. Almost all were from high income countries, although a number explored the perspectives of deprived communities within those countries. There was a wide variation in the health‐care service provision context, particularly in relation to payment for services through insurance or direct methods.

### 3.2 Underlying mechanisms for urgency

**Figure 2:**
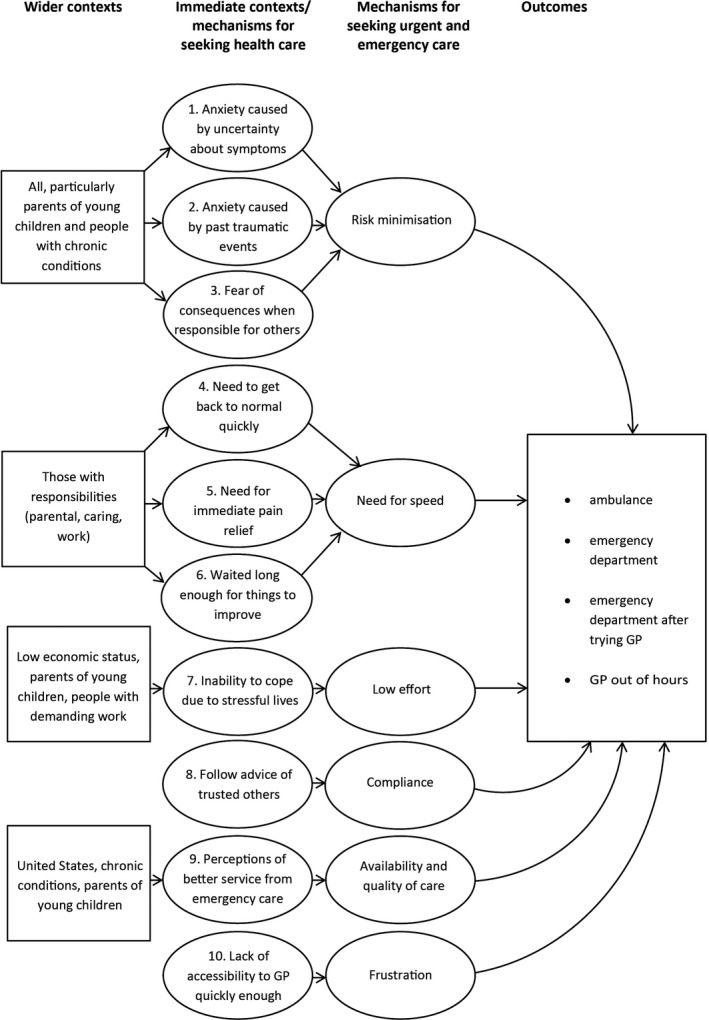
Overview of contexts and mechanisms affecting use of emergency and urgent care

Figure 2 provides an overview of the six underlying mechanisms for urgency of help‐seeking. The first was ‘risk minimization’ where patients sometimes felt that their symptom posed a potential risk to their health and sought health care quickly to minimize risk to themselves or others. Three programme theories shared this underlying mechanism. The second was ‘need for speed’ where patients were sometimes unwilling to wait for a routine appointment because they wanted the problem sorted out immediately. Three programme theories shared this underlying mechanism. The third was ‘low effort required for help‐seeking’ where patients sometimes accessed services which presented the lowest effort because their lives were complex or stressful. One programme theory had this underlying mechanism. The fourth was ‘compliance’ where patients sometimes followed the advice of trusted others about seeking help, or where to seek it from, rather than make a decision by themselves. Compliance is a term associated with following the advice of health professionals. We chose it here to also include following the advice of family or friends because some patients described doing what a family member told them to do as well as seeking advice from lay networks. One programme theory had this underlying mechanism. The fifth was ‘availability and quality of care’ where patients were sometimes attracted to attributes of emergency services. One programme theory had this underlying mechanism. The final one was ‘frustration with access to GP’, where patients sometimes felt frustrated because they could not get a GP appointment within their desired timeframe, or believed that it was not possible to obtain a GP appointment in a timely manner. One programme theory had this underlying mechanism.

### 3.3 Programme theories

We identified ten 10 interrelated programme theories proposing explanations for patient behaviour (Figure 2). We describe the programme theories in detail, along with the population subgroups associated with the programme theory (Table 1). We detail the qualitative evidence used to identify and refine each programme theory, links to existing theory, and the quantitative research used to test each programme theory (Table 2).

**Table 1:** Detailed programme theories

Programme theory (PT) label	Programme theory detail	Subgroups most relevant to
PT1. Uncertainty about symptoms causing anxiety	When there is uncertainty surrounding symptoms (M) either because they do not fit with people's expectations or prior experience (eg last longer, are more severe, unfamiliar or do not respond to self‐care in the expected timescale) (C/M), this increases the perceived risk that the problem may be serious (M) and an immediate need to establish what is wrong and obtain reassurance (M). This concern prompts the use of the ED (O), where it is perceived the most appropriate resources and expertise required to establish cause can be accessed quickly (C), often in the context of timely or satisfactory answers not having been received from primary care services (C).	
PT2. Heightened awareness of risk as a result of experience or knowledge of traumatic health events leading to anxiety	When people have experience of previous traumatic health incidents (eg delayed help‐seeking leading to serious consequences), or awareness of such incidents experienced by others or in the media (C), they have increased anxiety and awareness of danger (C/M) and reduced confidence in their own judgement (M). They are therefore unwilling to take risks when a health problem arises (M), leading them to seek immediate help and advice from an expert in the form of emergency care including ambulance services and EDs (O).	
PT3. Fear of consequences when responsible for others	When people are in a position of responsibility for others they are less willing to take risks with someone else's health than with their own and fear the consequences (eg distress/guilt, dismissal, litigation) (M) of not doing ‘the right thing’. This leads them to seek or to recommend seeking urgent care, particularly the ED (O).	Parents of a child, carers of vulnerable elderly people, people with chronic conditions, health services or other service professionals, for example teachers
PT4. Inability to get on with daily life	When people are prevented them from undertaking their normal lives, roles or responsibilities (eg paid work, childcare) (C) this creates a need to get back to normal quickly (M), to get on with their lives and discharge their responsibilities. This prompts use of urgent care (O) because it can resolve a problem quickly by being both more accessible and efficient than alternatives (C).	parents of young children, people working in jobs where they cannot afford to take time off or it is difficult to take time off
PT5. Need for immediate pain relief	When people are in pain or discomfort which they find intolerable (C/M), and they believe or experience that no primary care appointments are available within an acceptable time period (C), they seek care from a more urgent service—usually the ED (O)—because of a need to obtain prompt relief from their distress (M).	
PT6. Waited long enough for things to improve	When people delay seeking primary care treatment (for various reasons including deliberation and indecision, cost of treatment, lack of transport, complex living situations, mistrust of health services and work responsibilities) (C) they wait, often using self‐help measures, and hope the situation will improve or go away (C). The condition reaches a ‘tipping point’ where either it is no longer tolerable (M) or other circumstances force a decision (M), and people feel they cannot wait any longer (M). At this point, if a primary care service is unavailable to them (C), they feel they have no choice but to use an emergency service (O).	
PT7. Stressful lives/ can't cope	When people are already experiencing significant stresses which impact on the internal and external resources available to them (money, time) (C) they have less capacity to cope with the additional challenge of a new or changed health problem. Symptoms are therefore likely to trigger emotional distress, including feelings of loss of control and helplessness (M), leading them to use emergency services because this is less burdensome than making an appointment with a GP. This is more likely to occur when people cannot easily or quickly access a primary care service (C).	low socio‐economic status, parents of a child, isolation, demanding work, mental health problems
PT8. Following advice of trusted others	When people are anxious or concerned about a health problem and have sought the advice of trusted others (C)—either in their social network (eg family) or health professionals (particularly primary care staff)—and have been advised to seek urgent care, particularly the ED (M), they are likely to then use those emergency services (O).	
PT9. Perceptions or prior experiences of services	When people have individual experience or knowledge, or cultural beliefs, about the differing quality or availability of primary and emergency services (eg primary care offering inadequate diagnosis and care or discrimination (US context only), or EDs having better resources, expertise or more thorough care (C), they are likely to choose emergency care, particularly the ED (O) in which they have more trust and confidence (M).	people previously referred to emergency services by primary care staff, parents with young children, chronic conditions
PT10. Poor access to a GP	When people are unable to obtain an appointment with a primary care practitioner (C/M) this can further exacerbate the feelings of anxiety and cause panic (M). Individuals can experience feelings of frustration (M), mistrust (M), and the perception of an uncaring service (M), feeling they have no other choice (M) but to contact an emergency service (O).	

**Table 2:** Evidence for each programme theory

Programme theory	Qualitative research	Existing theory	Quantitative research
1. Uncertainty about symptoms causing anxiety: I am worried because I do not know what is wrong	A decision to seek emergency or urgent care seemed likely when there was uncertainty surrounding the symptoms. This uncertainty manifested itself in various ways: where the cause of the symptoms were unknown,^32^, ^33^, ^36^ the symptoms were ‘different’ or more severe than previously experienced,^37^, ^38^ or symptoms lasted longer than expected.^36^, ^39^, ^40^, ^41^, ^42^, ^43^, ^44^ This uncertainty surrounding symptoms could increase the perception of risk that there might be something seriously wrong.^36^, ^37^, ^39^, ^40^, ^45^ This created a need for fears to be allayed by seeking reassurance that the problem was not serious and that the illness was being treated appropriately.^33^, ^39^, ^42^, ^46^, ^47^, ^48^ *‘I do not know what I have, but it worried me, so I preferred to come immediately to the [emergency department] so at least I am reassured’.* 33 ^(p5)^ Uncertainty surrounding the cause of symptoms, and the need for reassurance, was particularly prevalent amongst parents of young children^42^, ^46^, ^47^, ^48^ who often have to rely on signs and behaviours of their children to ascertain what was wrong.^39^, ^41^, ^42^, ^44^, ^45^, ^47^ *Children can't tell you what's wrong, and parents want to make sure everything is OK* 41 ^(p1099)^	There was considerable support for this programme theory from existing theories, as well as further understanding of how anxiety affects decision making. Leventhal's Common Sense Model^49^, ^50^ suggests that when experiencing symptoms, people form a ‘cognitive representation’ of their illness based on knowledge and experience. This representation is comprised of the identity, duration, cause, controllability and consequences of the symptoms and is used to determine the amount of threat it imposes and therefore what coping strategies or other help‐seeking action should be taken. Leventhal highlights that help‐seeking is more likely to be triggered when people are unable to fit their symptoms to a label, or when their initial identification is disrupted due to the symptoms unexpectedly changing or continuing. The role of uncertainty in decision making has been explored, defined as the inability to determine the meaning of illness‐related events or to accurately predict their outcome.^51^, ^52^ This can be due to a range of factors including lack of clarity in the symptom pattern, unfamiliarity of symptoms, or inconsistency with expectations. In addition, illness and pain have been found to impact on people's information processing, undermining their ability to make sense of their illness, further increasing uncertainty.^51^ In situations of uncertainty, coping ability decreases, whilst anxiety and a sense of threat are increased, all of which are likely to increase help‐seeking behaviour. Cameron highlights how anxiety is associated with more impulsive, habitual patterns of behaviour, less ability to identify alternative strategies of action and reduced capacity to take in advice and information.^53^	There was evidence from cross‐sectional surveys of service users at emergency departments and GP out of hours services that attendees were worried or anxious^29^, ^31^, ^54^ or perceived their problem to be serious.^26^, ^55^, ^56^ There was evidence that a feeling of helplessness was also an important mechanism for parents of young children.^54^ Surveys also highlighted that not all users expressed anxiety or thought their problem was serious.^54^, ^56^
2. Heightened awareness of risk as a result of experience or knowledge of traumatic health events leading to anxiety: After what happened before I don't dare risk it, I don't trust myself	The importance of past experiences and how these affected decision making was evident in the literature. There were indications that individuals were more likely to be anxious and more risk averse when they had experienced a traumatic event in the past,^38^, ^42^, ^43^, ^48^, ^57^, ^58^, ^59^ had experienced an occasion when the illness had been more serious than they first thought,^44^, ^58^ were aware of the adverse experiences of others,^46^, ^59^ or media campaigns/news stories had heightened awareness of potentially life‐threatening conditions.^45^ *Since this incident a decade ago (which resulted in a bypass), the patient felt ‘as far as my heart's concerned, there never is any hesitation anymore’ […]‘Because of the previous heart [problems], I know it was ten, eleven years ago, but, I get very anxious when things start to happen with my heart and I like to get it seen to straight away’. (He called an ambulance immediately)* 58 ^(p338)^ This experience or knowledge resulted in heightened awareness leading to a concern or belief that the illness could be a threat to life,^38^, ^44^, ^48^, ^58^, ^59^ a tendency to be over‐cautious, and fear and anxiety arising at the slightest of symptoms.^43^, ^48^, ^58^ Past incidents could have a subconscious effect.^48^ *‘During the study interview, Ms S was asked about any prior experiences she might have had with the [paediatric emergency department], and she recalled that she had herself presented to the [emergency department] with severe abdominal pain, subsequently diagnosed as an ovarian cyst. In what can only be described as a ‘light bulb’ moment, Ms S's face shone with sudden insight as she connected her own experience with abdominal pain to her anxieties about her daughter’*.^48^ ^(p24)^ A traumatic incident in the past could lead to a loss of confidence and feelings of helplessness in their ability to diagnose and manage the illness, particularly for parents of young children^42^, ^43^, ^44^ who were considered to be more vulnerable.^42^ Fear or psychological distress created and increased the need to get help as quickly as possible^60^, ^61^ and a need to hand over the decision making to somebody with more expertise.^43^, ^58^ The psychological effect of a past health scare could also be seen in those with chronic conditions^38^, ^44^, ^58^ who were more likely to have experienced significant health events.^44^, ^57^, ^59^	There was considerable support for this programme theory from existing theories. As described in the previous section, Leventhal's Common Sense Model can be used to understand how people use their present moment experience and accumulated knowledge and beliefs to interpret their symptoms and decide on a course of action.^49^, ^50^ One key influence on these decisions is personal experience of a prior traumatic or life‐threatening event, or knowledge or awareness of such experiences in others in their social network. Once a situation is perceived as threatening, anxiety increases and Cameron identifies how an increased perception of danger prompts selection of risk‐averse options as well as a desire for diagnostic tests and a belief in their benefits.^53^ Even when no direct experience is present, Leventhal notes how the media can inform representations of illness,^49^ whilst Pescosolido emphasizes the influence of social norms in perceptions of illness and response to it.^62^ In this context, Beck's work on the ‘Risk Society’ suggests that people are operating within a risk‐based culture, which places emphasis on the responsibility to prevent problems before they arise.^63^, ^64^ Such a future‐orientated perspective and concern to avoid blame is likely to trigger early help‐seeking behaviour.	None of the included quantitative research considered the effect of past traumatic events. As noted in the qualitative evidence, this issue may be something that is not necessarily apparent to the individual so may not be amenable to quantitative testing.
3. Fear of consequences when responsible for others: In my position, it's better to be safe than sorry	The concept of ‘caretaker responsibility’ was specifically reported by Guttman^41^ who noted that the notion of responsibility was used as an explanation by parents in situations which they clearly did not equate with a medical emergency even though they were visiting a paediatric emergency department. Those with responsibility for making a decision on behalf of others seemed to be less tolerant of risk and more likely to err on the side of caution. This lower tolerance of risk and a ‘better safe than sorry’ attitude was implicit in much of the paediatric literature^41^, ^42^, ^43^, ^45^, ^46^, ^47^ and was related to feelings by parents of having a ‘duty of care’ to provide the best possible care to relieve any suffering. It was for this reason that expert opinion was often sought either at the paediatric emergency department^42^ or the GP out of hours.^43^ *‘[I'd] rather be safe than sorry’ […]‘I am a mother’* 46 ^(p221)^ *‘sometimes it just overwhelms me…I just feel what if I missed something, if anything happened I would feel the weight on my shoulders’.* 43 ^(p237)^ This low tolerance of risk was enacted under a societal expectation that risks should not be taken with a child's health^36^ and was endorsed by practitioners who stated that they preferred to trust parents' instincts and refer to the emergency department rather than risk a child's health.^47^ It was not only the consequences relating to the illness of the cared‐for person that were feared but also the feelings of distress and guilt that would result from not pursuing the best possible care.^41^, ^65^ This created an additional dilemma of balancing the guilt of not doing enough against that of being an unnecessary burden on emergency services.^43^ *Carers may feel responsibility to take an optimal and least risky course of action for their cared for in a perceived health emergency. Informal carers reported feelings of helplessness and wanting to avoid a situation of feeling guilty for not doing enough* 65 ^(p451)^ Although caretaker responsibility and having a duty of care were predominantly witnessed in the paediatric literature, this were also seen in relation to those responsible for elderly people and people with complex medical problems^65^, ^66^ and people in positions of responsibility such as teachers, employers, the police. Calnan et al^36^ found that there was a greater likelihood of a decision being made to seek help from the emergency department rather than general practice when that decision was made outside the home by people other than the individual or their relatives. In these instances, it was argued that the driver for the decision was the potential moral and legal consequences of not acting in the way commonly regarded as being appropriate.^36^	There was considerable support for this programme theory from existing theories. Leventhal identified that one of the important considerations of a person's self‐regulation of their health and coping behaviour was an assessment of the likely long‐term consequences.^53^ For parents and carers of vulnerable people the consequences of ‘doing the wrong thing’, that is not seeking help, could be both devastating and profound, both for the sick individual and the person responsible for their care. This sense of responsibility is increased within the ‘risk society’,^64^ with the increasing risk of legal action when mistakes are made and public scrutiny of the morality of individuals' decisions. In this context, social norms of caution predominate. Dixon‐Woods^72^ found that carrying ‘responsibility for others’ over‐rode a person's consideration of being ‘undeserving’, such that those who were responsible for the welfare of others (partners, elderly parents and children) felt an explicit sense of entitlement which justified ‘being demanding’. Dingwall noted that in contrast to adults attending with trivial problems, emergency department staff did not apply the same categorization of ‘bad patients’ to children brought for treatment, and that social norms meant they were automatically upgraded to ‘mandatory preciousness’.^73^	There was little evidence exploring this in the included quantitative research. There were tenuous links in that autistic children had higher rates of non‐urgent use of emergency departments.^74^
4. *Inability to get on with daily life: I need to get back to normal*	*Evidence from qualitative research*: The need to be able to function and get on with everyday life was found to influence whether a person sought urgent care,^37^, ^67^ most often in relation to work and/or child care responsibilities. The need to take care of social responsibilities meant that individuals used an emergency service at a point when they no longer felt physically able to discharge their responsibilities; this particularly related to looking after children. Stafford^37^ identified how the inability to perform these activities of daily living resulted in distress which motivated individuals to seek urgent care. *I called my Mom on Monday because I was in so much pain. And well anyway, I have a little baby and I really can't take care of him real well and I was at home by myself.* 67 ^(p558)^ *Support from existing theories*: There was considerable support for this programme theory from existing theories. Both the Illness Action Model and Common Sense Model of behaviour^50^, ^53^, ^68^ propose that when faced with illness, individuals take action to regulate or manage threats to normality in physical and social functioning. Leventhal identifies the consequences of illness, including impact on function, as one of the key domains of illness representation,^50^ and Cameron's work using this model found the degree of disruption experienced due to symptoms to be an important trigger for help‐seeking.^53^ Interference with sleep has been found to be a significant influence in this context.^69^ Zola^70^ identifies five triggers for help‐seeking, including perceived interference with vocational or physical activity and perceived interference with social or personal relations and suggests that these factors are potentially more important than the stress of the illness itself in prompting help‐seeking. Underlying social and cultural norms will also significantly influence norms of behaviour. Zola highlights cultural differences in the significance of particular triggers to help‐seeking,^70^, ^71^ whilst Beck suggests that a social emphasis on individual responsibility encourages people to take action to maintain their health and working ability in order to avoid blame.^63^, ^64^ *Support from quantitative research:* This issue was not addressed in the included quantitative research. It is possible that it is labelled as convenience use of emergency and urgent care in this literature.^28^		
5. Need for immediate pain relief: It's urgent because it hurts	The need for relief from pain as quickly as possible was prominent within the qualitative literature.^33^, ^37^, ^41^ Pain was not necessarily considered an emergency in terms of being ‘life‐threatening’0.^41^, ^75^ Rather there was a perceived need for urgent or fast care, sometimes defined as an emergency, to ease the pain and the distress it was causing.^32^, ^33^, ^37^, ^41^, ^47^, ^58^, ^60^, ^67^, ^75^, ^76^, ^77^ *Pain intensity, and associated with this, a desire for quick relief of pain, was a key driver for seeking urgent care: ‘The pain, it was just, I've never felt pain like that before’ ‘I was in so much pain…it was so intense… it was just too much…I was so desperate for some relief…I have a child and labour's meant to be painful but (not) compared to that’.* 37 ^(p68)^ Caretakers found children in pain intolerable^41^, ^76^, ^78^ and Guttman^41^ found this to be one of the primary reasons for using a paediatric emergency department. This behaviour was associated with uncertainty about symptoms (programme theory 1), parental responsibility (programme theory 3) and ability to function, such as eating, sleeping and working (programme theory 4). It was sometimes reported that the person had first attempted to gain an appointment with their GP when experiencing pain^33^, ^37^, ^67^ and it was only when a timely appointment was unavailable that an emergency service, primarily the emergency department, was used.	Leventhal's Common Sense Model^50^ suggests that pain or other symptoms trigger the development of a ‘cognitive representation’ or interpretation of the situation which then guides the individual's action. The model identifies one key dimension of this representation as the controllability of the symptoms and in a situation where pain is experienced as unmanageable, this is likely to trigger help‐seeking action. Leventhal also recognizes the significance of the emotional response to symptoms. In this context, Cameron notes that anxiety has been found to increase the painfulness of symptoms,^53^ which in turn is likely to further impact on anxiety, and thus on the mechanisms of decision making identified in programme theories 1 to 3. In relation to Andersen's model of health‐care utilization, Hodgins and Wuest^28^ found that severity of symptoms was a key reason given for emergency department use, with less willingness to wait being particularly associated with pain and injury. The social dimension of this is illustrated by Beck, who proposes that the development of a culture which promotes medicine as the solution to problems has led to reduction in the tolerance of pain or illness.^64^	The need for pain relief was not addressed within the included quantitative research.
6. Waited long enough for things to improve: I can't delay this any longer, I need to deal with it now	People described delaying seeking care, and would ‘wait and see’, often using self‐care methods, before accessing emergency services.^32^, ^33^, ^40^, ^57^, ^67^, ^75^ Reasons given for such a delay were varied and included a belief or hope that the problem would resolve itself over time, or deliberation and indecision about using primary care services appropriately or mistrust of the medical authorities.^79^ Complex and difficult living situations, particularly experienced by those with low economic status, could mean that dealing with day to day challenges (financial, employment, child care) took priority over health care.^57^, ^76^, ^79^ Additionally, due to work commitments during the day, decisions to seek care would often not be made until the evening when symptoms had deteriorated and/or anxieties increased, particularly for parents of children with fever.^39^, ^78^ *‘You thought it was an emergency? How did you decide it was an emergency’?* *P6: ‘Because I'm feeling a lot of pain, (barely audible) for six weeks’* 77 One consequence of these delays was that help was only sought when the problem became physically or psychologically intolerable and there was then a need to get help quickly. Once people had waited and deliberated for some time, they made a decision that they had waited long enough and any further delay could not be endured.^39^, ^44^, ^60^ However, a timely primary care appointment might not be available,^57^, ^66^ leaving only the choice to attend an emergency department or contact an out of hours service. This was primarily reported in the parent/child literature.^39^, ^44^ *‘Parents generally cautiously wait and see before contacting GP out‐of‐hours care. When they decide to seek care many stated that nothing could persuade them from wanting to see a doctor at that point and that was their main reason for contacting the GP out‐of‐hours centre and not their own GP “nobody could have said to me: no, you do not need to come over right now, just visit your own GP tomorrow”’* 39 ^(p4)^	There was considerable support for this programme theory from existing theories. The duration of symptoms is identified as a key predictor of help‐seeking in both Andersen^80^, ^81^ and Leventhal's^50^ work, whilst Mishel recognizes how unexpected duration contributes to uncertainty and therefore influences decision making.^51^, ^52^ Leventhal's Common Sense Model suggests that there is a period of delay between the onset of symptoms and seeking help, during which the person appraises the symptoms and addresses the situation using ‘active problem‐solving behaviours’0.^49^, ^50^ It is only when their appraisal is challenged by symptoms continuing or worsening despite their actions that people interpret it as serious and seek help. This understanding is supported by Rogers et al who identify that, in most illness episodes, no external help is sought at all and the situation is managed through self‐care or waiting for it to resolve.^82^ Symptom duration is noted as one of the key triggers for finally seeking professional care (also identified in Programme Theory 1 as increasing uncertainty and anxiety), along with impact on function identified in Programme Theory 4 and coping capacity identified in Programme Theory 7. There is also a strong social dimension to delays in help‐seeking, with Zola identifying how people from different ethnic groups were eventually prompted into help‐seeking behaviour by a range of triggers.^70^, ^71^ One such trigger, ‘temporalizing’, where people decided to wait for a specified amount of time, was particularly associated with Anglo‐Saxon Protestant patients but did not significantly influence other groups.	There was considerable support from cross‐sectional quantitative studies for people delaying attending services and trying to self‐manage problems: there was an increase over time in emergency department users who had waited a week or more before attending^23^; duration of symptoms was an issue for emergency department users^55^, ^83^; a survey of people with minor injuries in an emergency department identified a delay in help‐seeking^31^; 68% of people in an emergency department waiting room^28^ and 21% of febrile children attending a GP out of hours service^54^ had used over the counter remedies beforehand; people with illness waited longer than people with injury before attending an emergency department^84^;and 41% of non‐injuries in a paediatric emergency department arrived 2‐7 days after onset.^56^ In a comparative study, medically unnecessary users of GP out of hours had longer lasting problems than medically necessary users.^29^
7. Stressful lives: I just can't cope with the illness or making an appointment	The theme of distress and its impact on the use of emergency services was most evident in research conducted in populations of low socio‐economic status.^57^, ^59^, ^67^ Stressors experienced were of both a social and psychological nature included social isolation and limited social networks,^43^, ^59^, ^67^ single parentage,^43^, ^57^ problems with family and social relationships,^67^ grieving,^67^ housing and financial difficulties,^57^, ^59^, ^75^ being unable to afford to take time off work,^46^, ^48^, ^61^, ^76^ discrimination^57^, ^75^ and the traumatizing impact and disruption to life and work of long‐term medical problems.^59^ Implicit within this literature was that those dealing with distress in their daily lives had fewer material, social and health resources available to them, the absence of which were stressors in themselves. People thus had multiple responsibilities to manage with too few resources.^48^ Current levels of stress were often associated with past trauma of either a medical or non‐medical nature (see programme theory 2). Olsson et al^59^ noted how most participants had ‘struggled hard throughout their lives’ and highlighted the amount of ‘threat’ and ‘danger’ that featured in the narratives. Although they may make concerted efforts to cope, feelings of loss of control and helplessness lead them to seek emergency care. The emergency department was often accessed due to ease of use which was important in the context of stressful lives^48^ and could be regarded as a place of refuge and safety in times of distress.^67^ *Erik, now divorced and isolated, talked about his episodes of headache as a suffering similar to what he felt five years earlier when his head was injured as a result of assault. He is very anxious that the after effects of his injury will eventually lead to his death ‘That feeling of impending doom, that fluttery feeling in your chest, I felt I was losing ground, so to speak .. I get twinges in my chest, I was almost dying … I have no‐one who can sound the alarm or help me, so I went [to the emergency department]’* 59 ^(p432)^ The perceived lack of social and health service support, particularly during the night, combined to increase people's feelings of vulnerability and stress.^43^, ^78^ Increased levels of anxiety and tiredness further hampered the ability to think rationally. Parents of young children were found to make frequent use of GP out of hours services at night,^43^ whilst people with complex stressful lives and with little social support were reported as frequent users of emergency services.^43^, ^57^, ^59^ *‘Night times are the worst…During the day, I think you can be more rational about it, but it gets to night time and obviously symptoms usually get worse at night don't they,… and you just, you start to panic a bit more because you're tired, they're tired and you don't have your wits about you as much, I think’.* 78 ^(p5)^ People in distress could view the process of seeking a GP appointment as burdensome (see Programme Theory 10), or difficult to access due to financial difficulties or lack of transport.^32^, ^57^, ^65^, ^75^ *Support from existing theories*:	There was considerable support for this programme theory from existing theories. Andersen identifies lack of coping capacity as a predisposing factor to health‐care utilization,^80^, ^81^ and Antonovsky highlights how coping is linked not just to the problem being faced, but to the resources available to a person to manage it.^85^, ^86^ The latter identifies a range of ‘Generalized Resistance Resources’ or characteristics which help people manage stressful situations including physical (eg health), material, cognitive and emotional, social support, and attitudes and coping styles. Antonovsky suggests that the availability of these resources impact on a person's tendency to see their life as more or less ordered, predictable and manageable, labelled as a ‘sense of coherence’. Those with a weak sense of coherence are less resilient and more likely to see stressful situations as threatening and anxiety provoking.^85^ Feelings of lack of control create helplessness, resulting in an inability to use the resources available and thus a reduction in coping capacity. The ‘candidacy theory suggests that in these circumstances people are likely to use services which present the least barriers to access.^72^ Gaining access to health care can be complex, and those who lack resources and competencies are likely to opt for more ‘permeable’ services, for example those which do not require appointments or a need to clearly articulate a problem in order to access help. In addition to the impact of anxiety discussed in programme theories 1 and 2, other work highlights how stresses including illness or time and resource constraints impact on the decision‐making process in a variety of ways. These include reduced sense‐making and problem‐solving ability,^51^, ^52^ increased likelihood of more spontaneous and less considered decisions,^62^, ^87^ and a greater sense of urgency and narrowing of focus to meet immediate short‐term needs.^88^, ^89^	There was some support for this in the included quantitative articles: people arriving at an emergency department by ambulance and classed as non‐urgent were more likely to be homeless and have mental health problems^33^; people who were more likely to use an ambulance in a hypothetical situation that did not require an ambulance had no car or they lived alone^6^; and 49% of parents of a febrile child who used a GP out of hours services felt helpless.^54^ However, not all the studies supported this programme theory. One review concluded there was little evidence for the association between personality, including coping mechanisms, and use of emergency departments^4^ and another that there was some evidence that affluent groups were more likely to go to an emergency department for minor problems.^83^
8. Following advice of trusted others: That's what they said to do, and they know better than me	The involvement of others in decision making to contact an emergency service was a strong theme that ran through much of the literature, particularly relating to emergency departments. This could be advice received either from family and friends^32^, ^36^, ^39^, ^40^, ^44^, ^46^, ^65^, ^67^, ^77^, ^90^ or from primary care services.^32^, ^47^, ^48^, ^61^, ^67^, ^77^, ^90^, ^91^ McGuigan et al^40^ stated that being advised by others, particularly family, was a common reason for deciding to attend the emergency department, with a tendency for advice to be sought by women; they referred to this as ‘sanctioning’. Research conducted in the Caribbean found that use of the emergency department was a socially shared custom in which family and friends encouraged the habitual use of the service,^77^ indicating cultural as well as individual mechanisms at play. Contacting trusted friends and family was conceived as a natural coping mechanism when in distress and indecisive about what to do.^37^, ^67^ Additionally, when feeling in distress, people were more likely to be receptive to the advice of another.^37^ *‘I spoke to my mother about it. And um, she actually brought me to the emergency department. She said my Dad had the same thing and it was just, it was polyps or something … but she said I should probably come in’.* 67 ^(p588)^ Whilst there was evidence that family and friends had the most impact on decisions regarding the use of emergency services,^36^, ^40^, ^58^, ^90^ health‐care practitioners were also influential in this decision‐making process. Primary care practitioners and staff were often described as having recommended attendance at an emergency department for both adults^32^, ^67^, ^77^, ^90^, ^91^ and children.^47^, ^48^, ^61^ Individuals were essentially given permission to attend an emergency department,^41^, ^58^ with the decision being sanctioned by another ‘trusted decision maker’0.^41^, ^58^ This in turn could influence and encourage future use of emergency services in similar circumstances to save time and ‘cut out the middle man’.^58^ *‘The GP would probably have just sent you to the hospital anyway…At our place they do it with [everything], if they don't know enough they just send you straight to the hospital’.* *‘(The second time) I just drove to the hospital, I thought I'm not even messing about going there [to the GP]…I'll just go straight to the hospital’* 37 ^(p69)^ There was evidence to suggest that, once advised to take this course of action, there were feelings of obligation to do so, even if it was not considered appropriate, particularly where children were concerned (see Programme Theory 3). It was also noted that individuals may have felt pressurized by others into contacting an emergency service when they would not have ordinarily done so.^36^ In turn, the advice given by others may be influenced by their perceived moral and legal obligations and thus they advise the least risky course of action (see Programme Theory 3).	There was considerable support for this programme theory from existing theories. Social and cultural influences on health behaviour have received greater acknowledgement in later formulations of both Leventhal's Common Sense Model^49^, ^50^ and Andersen's Model of Health‐care Utilization.^80^, ^81^ Pescosolido^62^ places the most emphasis on the social context in which people operate, highlighting how illness beliefs and behaviours are influenced both by individual social networks and the social structure. These influences can be both direct and indirect. Direct influence takes the form of being advised on a particular course of action by peers or by health‐care professionals, with some groups having more ready access to the latter due to their social and economic characteristics.^72^ Pescosolido also notes how people are most likely to adopt a behaviour if they know others are doing the same, particularly when those people are similar to themselves and that those in dense social networks appear more likely to delay help‐seeking, but people are more likely to have an ‘avoidable visit’ if they have consulted family members.^62^, ^87^	Cross‐sectional quantitative studies offered evidence to support people following the advice of family and friends, and of health professionals—especially general practice—when attending an emergency department. The most frequent reason reported for attending an emergency department was following the advice of others.^30^ Family and friends offered advice to 31% of emergency department attenders,^24^ and 52% had discussed their febrile child with others before calling a GP out of hours.^54^ It was also common to follow instructions from primary care staff: 27%,^25^ 26% 24 and 66%^56^ were referred to an emergency department by a GP, with the proportion unclear in other studies.^4^, ^31^, ^55^ There was also some evidence that people went straight to the emergency department because they felt the GP would send them there anyway.^31^
9. Perceptions or prior experiences of services: I'll get a better and faster service from the hospital/ambulance	Emergency services were often accessed because people believed that primary care lacked the necessary care, expertise or resources to provide good quality care. This could be due to a generally held belief, sometimes acquired from family and friends,^32^, ^65^, ^77^ or based on past personal experience.^36^, ^41^, ^42^, ^46^, ^47^, ^57^, ^61^, ^65^, ^66^, ^76^, ^77^, ^91^, ^92^ Patients reported being dissatisfied with general practice for a range of reasons: perceived inadequate care or misdiagnosis^36^, ^41^, ^47^, ^66^, ^77^, ^78^, ^92^; the short amount of time spent with the doctor and/or lack of thorough examination, particularly where children were concerned^42^, ^46^, ^61^, ^77^, ^78^, ^91^; not feeling listened to^41^, ^57^; failure to answer questions^47^, ^78^; or not being taken seriously.^41^, ^57^ Such experiences could lead to a lack of confidence and trust in general practice^41^, ^47^, ^66^, ^76^, ^78^, ^79^ and the use of an emergency service, particularly emergency departments, to obtain a second opinion.^36^, ^41^, ^46^, ^57^ *a mother of a two‐year‐old did not like what she was told the day before by the doctor at the clinic. Apparently he did not offer a good explanation of the diagnosis of the child's condition. She was still scared and felt she was not giving her child the right medication. She came to the [emergency department] to get a ‘second opinion’ and a better explanation* 41 ^(p1100)^ Conversely, emergency departments were accessed because of a belief that they were the best or most appropriate place to be due to the availability of expertise or resources such as laboratory tests, X‐rays, etc^33^, ^40^, ^41^, ^42^, ^46^, ^47^, ^61^, ^66^, ^75^, ^76^, ^77^ Again, this perception could be due to past personal experience or a culturally held belief perpetuated by friends and family. Having both access to resources and the expertise of emergency department practitioners meant that patients had trust and confidence in the service and hence felt safe.^41^, ^58^, ^76^ This seemed to be especially true for parents^42^, ^46^, ^47^, ^61^, ^76^, ^78^ and for those with chronic conditions^58^, ^90^ where familiarity and previous experience played a part in the decision making when feeling anxious.^41^, ^58^, ^66^ *Mother 1: ‘I feel that the [emergency department] doctors are more skilled’.* *Mother 2: ‘They do a better check‐up and they give them better medicine. Here they look at him, they weigh him, they look at his eyes, his throat, they take his blood pressure, they check his little heart, his lungs, and they examine him like I like them to examine him, to really know what problem he has’.* 61 ^(p364)^ *‘In hospital they've got everything there, they've got the ventilators, the drips, they've got everything, they can resuscitate you, if need be […] I feel safe going in a hospital’.* 58 ^(p338)^	There was considerable support for this programme theory from existing theories. ‘Recursivity’, or the influence of past experiences of services on patterns of future use, can result in poor experiences of care either reducing use of a service or increasing it in the desire to obtain resolution of a problem.^82^ Andersen identifies satisfaction with services as predisposing factor to health‐care utilization, and recursivity is introduced as a key element of later iterations of the behavioural model.^80^ Other authors^93^, ^94^ also emphasize the role of habit within decision making, whereby once a pattern of behaviour is established, this is likely to continue, particularly where elements of the situation are familiar due to past experience. Experience may also come from sources other than direct contact with a service^95^ through ‘mediated experience’, where information is obtained and internalized from people's social network and media portrayals, and ‘imagined services’, where perceptions are based on wider cultural assumptions of the nature and quality of service provision. This is in line with Pescosolido's work, which argues that all actions are taken within a social context and cannot be understood without recognition of this.^62^ They identify how three systems interact to influence an individual's response to their symptoms: their individual social context (including social characteristics and prior experience of illness and services); their personal social network (including beliefs and attitudes, interactions with others for advice); and the treatment network (including the organization of health care and ease of access to treatment). In relation to the organization of services, their ‘permeability’ or ease of use impacts on people's decisions.^72^ In particular, those who are disadvantaged are likely to select services which are perceived to present least barriers to those with challenges such as low literacy, difficult time management or an inability to clearly articulate their needs.	There was considerable support from cross‐sectional quantitative articles for the attraction of the tests available and the quality of care at emergency departments. Some studies were vague by describing a belief that an emergency department was required 84 but others identified specific attractions of this service including providing a ‘one stop shop’ for people with chronic conditions,^96^ the ease of getting tests and treatments,^83^ the preference for a specialist within paediatric emergency departments^55^ and the availability of X‐ray facilities. X‐rays were a key issue in that around half of people attending an emergency department thought they might need one^24^, ^30^ or they were a reason why people perceived a GP would not be able to help.^25^ Further support for this was the belief that emergency departments were better than GPs for injuries.^31^, ^55^ Concerns about poor quality general practice were largely related to lack of tests such as X‐rays and accessibility (see Programme theory 10).
10. Access to a GP—I can't get an appointment quickly enough	The inability to obtain a timely appointment with a GP was a commonly reported reason for contacting an emergency service.^33^, ^37^, ^42^, ^61^, ^66^, ^67^, ^76^, ^91^ Whilst this could be a perception that they were unlikely to be able to get an appointment based on prior experience,^46^ it was commonly reported that people had first attempted to gain an appointment with their GP and it was only when a timely appointment could not be obtained that an emergency service, primarily an emergency department, was used.^33^, ^37^, ^42^, ^61^, ^66^, ^67^, ^76^, ^91^ *‘My doctor was on that day and she's part‐time and she's fabulous and so I rang five hours before she started work, but the receptionist said “she's booked out, you can't come in”’* 42 ^(p205)^ *In both the adult and pediatric interviews the issue of limited availability of timely appointments at regular place of care emerged as a recurring justification for the [emergency department visit]…A typical response was that ‘it takes too long to get an appointment at the clinic’. Some parents said that it takes two to three weeks to get an appointment, whereas others talked about wanting to get an appointment by the next day* 41 ^(p1103)^ There were indications that the inability to get an appointment when feeling ill and distressed could exacerbate existing feelings of anxiety and stress, leading to panic, and further increase the perceived need to get help quickly.^65^, ^66^, ^67^ High levels of anxiety could in turn exacerbate pain.^37^, ^60^ *The emergency number that the answering machine gave me re‐directed me back to the surgery and it just kept looping me around, so my ex‐husband, I think he just panicked and called an ambulance* 66 ^(p3)^ The inability to obtain an appointment and the complexity of appointment systems could lead to feelings of frustration and anger and an increased propensity to use an emergency service.^33^, ^37^, ^42^, ^47^, ^61^, ^65^, ^76^ This was particularly reported in the literature relating to parents with an ill child.^42^, ^47^, ^61^, ^76^ Frustration was also a factor amongst those with English as their second language who had difficulties communicating their requirements over the telephone when trying to get an appointment. In this situation people felt they had little choice but to make use of an emergency service, that it was ‘unavoidable’ and used as a ‘last resort’ because there was ‘nowhere else to go’^58^, ^65^, ^67^, ^76^ When feeling ill and in distress, with no timely GP appointment available, the emergency department was considered the most accessible service.^41^, ^58^ *One mother expressed frustration, because she had made an effort to ‘do the right thing’ and have her daughter seen at her [GP's] office but could not get a clear explanation of how to go about it.* 61 ^(p363)^	There was considerable support for this programme theory from existing theories. Anderson highlights how availability of services is a key enabling factor in people's utilization of health care, and in later work places increased emphasis on the role of differential access to services as a determinant of behaviour rather than the characteristics of the individual.^80^ This Programme Theory is a factor in many of the other Programme Theories and therefore many of the exiting theories discussed in the previous nine Programme Theories have relevance here.	There was evidence from cross‐sectional studies that perceived or actual difficulty accessing a GP in the time frame required by patients affected their use of emergency departments and GP out of hours services. This included unavailability of a GP,^24^, ^28^, ^31^ 19% being dissatisfied with GP appointments,^23^ negative perceptions of GP access,^4^ worse in‐hours access associated with GP out of hours use,^97^ difficulty accessing a GP in terms of getting an appointment,^83^ or not wanting to wait for a GP appointment for 12 hours or two days.^96^ Lack of access was sometimes due to the time of day, that is the primary care facility was closed,^98^ and unwillingness to wait for an appointment (see Programme Theory 6), as well as inability to get an appointment. In some studies, a sizeable minority of patients had attempted to contact the GP before going to an emergency department or GP out of hours service: 20% of those presenting in‐hours to an emergency department had been unable to get a GP appointment,^25^ and 25% had sought care from a GP.^84^ However, this percentage was lower in some studies: 8% reported poor access to a GP.^29^ Patients from deprived communities identified having more problems with access to a GP in working hours.^99^

## 4 DISCUSSION

### 4.1 Summary of findings

Six underlying mechanisms within 10 interrelated programme theories were identified to explain why patients made clinically unnecessary use of services providing emergency and urgent care: (a) need for risk minimization, caused by anxiety due to uncertainty about the seriousness of symptoms, by heightened anxiety due to past experiences of traumatic events, or by fear of consequences when making decisions about others, for example about children; (b) need for speed, caused by a need to return to normal to attend to responsibilities, a need for immediate pain relief, or because patients had waited for symptoms to improve and could wait no longer; (c) need for low treatment‐seeking burden, caused by inability to cope due to complex or stressful lives; (d) compliance, because family, friends or health services had advised such action; (e) consumer satisfaction, because emergency departments were perceived to offer the desired tests, expertise and ease of access when contrasted with primary care; (f) frustration, because patients had attempted and failed to obtain a GP appointment in the desired timeframe. It is likely that underlying mechanisms do not act in isolation but rather that a combination of mechanisms are likely to impact on an individual's care seeking behaviour. These programme theories were supported by existing theories on health behaviour and some were supported by quantitative evidence.

### 4.2 Context of other research

Some of the programme theories had been identified by the authors of the original reviews from which we drew our qualitative studies, although we were able to offer more understanding of how these issues affected people. In particular, uncertainty causing anxiety and the need to manage risk by getting reassurance^13^, ^14^, ^15^; fear of consequences particularly around children and the bystanders' role in use of ambulances^15^; stress and the need for low burden when seeking care in terms of social deprivation affecting ambulance use^15^; compliance in terms of other people recommending or making the decision to contact a service, including service providers^13^, ^14^, ^15^; consumer satisfaction in terms of positive views of emergency departments offering the expected investigations in a single place^13^, ^14^, ^15^ and negative views of general practice due to lack of confidence in GPs^13^, ^14^; and frustration around access to primary care.^13^, ^14^, ^15^ Some of our programme theories were also supported by research on general demand for emergency and urgent care. In particular, there was considerable support for the programme theory around poor access to GPs affecting use of emergency departments for the context of all users of emergency and urgent care rather than only clinically unnecessary use. Poor access to GPs was associated with higher use of emergency departments in numerous studies, including a large scale survey of GP patients in 31 countries.^22^

Some programme theories were not highlighted by the original reviews, in particular: the role of previous traumatic events; the need to seek immediate pain relief; the need to return to normal in order to attend to responsibilities; and the role of self‐imposed delay in creating urgency. This identifies the added value of this realist review.

Our programme theories did not include some issues which have been identified elsewhere: awareness of services,^23^, ^24^ although only 3% of people reported this as an issue in one study^24^; the convenience of the setting in terms of shorter distance to travel to an emergency department or GP out of hours service^14^, ^24^, ^25^, ^26^, ^27^; health knowledge^15^; geography in terms of rural and urban locations^28^; not having a GP^29^; patient misunderstanding of role of a service^9^, ^29^; the desire to take control through contacting a service^9^; lower cost/financial considerations^14^; and lack of transport.^13^, ^15^ These did not become programme theories because they did not appear strongly within the included qualitative literature of patients' perceptions of clinically unnecessary use. It may also be the case that there was subjective selection of issues within our study, although many detailed team discussions took place throughout our study to address this risk. Additionally, we did not have a programme theory around patients accessing emergency and urgent care because it was convenient, which was a key issue identified by other reviews and studies.^4^, ^13^, ^14^, ^30^, ^31^ In our review, this factor may have been represented through our programme theories on the need to get back to normal quickly to attend to responsibilities, and the impact of stressful lives creating the need for low treatment‐seeking burden.

### 4.3 Strengths and limitations

A key strength of our study was the time and care spent developing and refining the programme theories based on qualitative research with patients. A further strength was linking programme theories to existing theories of health behaviour. A key limitation was testing the theories within comparative quantitative studies. Although these studies were available, they did not measure some issues related to our programme theories.

Our realist approach identified similar findings to previous reviews but went further by examining reasons behind findings, for example exploring why people felt anxious. It also identified a number of new issues such as the need for immediate pain relief and the impact of previous traumatic experiences. There were some limitations to the review. First, included articles in the review focused predominantly on emergency departments, with a particular gap around use of daytime general practice, which is the most common first point of contact for those seeking urgent care.^1^ Second, the programme theories developed and refined here were based on qualitative interviews with patients who may present as ‘the rational me, the irrational other’ due to the moral dimension of help‐seeking behaviour,^8^ perhaps making circumstances sound more rational and justified than they were in practice. However, it is important to understand these presentations, and our review provides valuable insights into how patients describe their decision making. Third, participants in the included studies were selected for interview based on numerous different definitions of ‘clinically unnecessary’ and were not a consistently defined group, as is the case in other reviews. The inconsistency in how non‐urgency has been assessed in different studies has led to proportions of clinically unnecessary use varying between 4.8% and 90% depending on the definition and context.^5^ Fourth, the qualitative articles did not always offer enough detail to show how different issues interacted within individuals, or when a service was the first or last resort for interviewees. Finally, digital sources of health‐care advice are increasing in use and these did not feature in our findings possibly due to the age of the studies included.

### 4.4 Implications

The implications of our findings are that clinically unnecessary use of emergency and urgent care may be judged rational and reasonable once the details of each person's situation are understood. Indeed some of the research articles included here concluded that individuals appeared to behave rationally.^31^, ^32^, ^33^, ^34^ A potential intervention would involve education of policy makers and service providers in understanding patients' decision making. For example, if clinicians perceive that patients who have had symptoms for weeks do not require urgent care, then understanding that patients perceive they require urgent care precisely because they have had symptoms for a long time may change the judgements clinicians make. Having said this, in the context of demand outstripping the supply of many health services, a ‘population perspective’ rather than an ‘individual patient perspective’ suggests that interventions to change patients' practice may still be needed for the future sustainability of services.

A key finding was that service configuration and accessibility plays a key role in patients' decision making. Some patients try to take a route through the system of care that is commensurate with their clinical need by contacting their GP before attending an emergency department. However, it appears that GPs and other services (such as the urgent health‐care helpline NHS 111 operating in parts of the UK) advise patients to go to the emergency department, or patients are unable to obtain an appointment with a GP in their required time frame, or patients believe that they would not be able to obtain a timely GP appointment if they tried. Improving access to GPs might therefore alleviate some of the clinically unnecessary demand on emergency departments. Improving access to GPs might include increasing capacity in general practice. As highlighted in our review however, patients' decision making is driven by a complex interplay of mechanisms, and it is clear that improved GP access would not alleviate all clinically unnecessary demand because some patients feel strongly that they need the facilities offered by emergency departments, in particular X‐rays. Service reconfiguration may therefore be required in terms of offering X‐rays in places other than emergency departments.

A review of reviews of policy interventions to reduce use of emergency departments^35^ did not focus specifically on clinically unnecessary demand but identified six types of interventions to manage all demand: cost sharing, strengthening primary care, pre‐hospital diversion including telephone triage, coordination, education and self‐management support, and imposing barriers to access to emergency departments. Evidence of effectiveness of these interventions was found to be insufficient. However, the review authors pointed out that the most opportunity for improvement lay with ‘inappropriate’ visits to emergency departments. They highlighted the potential for testing co‐location of GPs in emergency departments and telephone triage systems. Although education and self‐management support is included as a policy intervention, no mention is made of societal level issues that may lead to inability to cope with even minor health problems. If meaningful changes in service use for people in these circumstances is to take place, these issues may need to be addressed through wider public health interventions, such as reducing poverty, improving support for child care and reducing stress caused by not being able, or not feeling able, to take time off work to seek health care.

Finally, there are likely to be groups of people who are habitually labelled as seeking clinically unnecessary use of emergency and urgent care, for example people who have difficulty coping. It is also likely that any individual may be labelled as making clinically unnecessary use of emergency and urgent care at some point in their lives because a specific symptom or circumstance causes high levels of anxiety for example. Interventions will need to consider both of these scenarios.

There is a need for further research. First, there is a need to standardize the definition of low‐urgency for specific services. Second, there is a need to further test the programme theories developed here by measuring the extent to which they explain clinically unnecessary use of care. Our team is undertaking a population survey measuring the propensity of people to make clinically unnecessary decisions in the context of hypothetical vignettes, where the programme theories can be tested and the size of effect of each determined. Third, there is a need to explore how these programme theories interact within individuals through further qualitative research that pays specific attention to these interactions. Fourth, there is a need to identify and evaluate interventions to address these programme theories. Evaluation of interventions is essential because they may have adverse consequences such as increasing demand for health care overall, or failing to offer cost‐effective alternatives to current practice. Finally, there is a need to explore why primary care staff recommend attendance at an emergency department to some patients who contact them.

## 5 CONCLUSIONS

Multiple interventions may be needed to reduce clinically unnecessary use of emergency and urgent care. These are likely to include changes to health service configuration and accessibility, and patients' social circumstances, rather than simply focus on individuals' behaviour.

## CONFLICT OF INTEREST

The authors declare that they have no conflicts of interest.

## Data Availability

All data generated or analysed during this study are included in this published article and its Supporting Information files.
