# Supplementary material for: ‘Clinically unnecessary’ use of emergency and urgent care: A realist review of patients' decision making
Source: Health Expect. 2019 Oct 29;23(1):19–40. doi: 10.1111/hex.12995 (PMC6978874; doi:10.1111/hex.12995)
Supplement: Supplementary file 1 [file HEX-23-19-s001.docx]

**Appendix 1 Description of included studies**

**QUALITATIVE STUDIES FOR DEVELOPING THE PROGRAMME THEORIES**

ED=emergency Departments, UCC= Urgent Care Centres, PED= Paediatric Emergency Departments, OOH= primary care out of hours services

| **Author, Year and Country** | **Service** | **Stated aim/objective, *plus additional text where this provides justification for inclusion of the paper*** | **Relevance** | **Data collection method; number of participants** | **Key themes/issues identified** |
| --- | --- | --- | --- | --- | --- |
| Agarwal et al  2011  UK | ED and UCC | To explore the reasons for attendance at the ED by patients who could have been managed in an alternative service | 1 | Semi-structured interviews;  23 adult patients and/or their carers | - *Anxiety*/concern re-presenting problem – belief it needed dealing with quickly, and *familiarity* with ED services - *Unable to access general practice*: no appointments, out of hours, too long to wait, difficult to get to - *Perceptions of efficacy of the ED*: already being treated there, more thorough investigation - *Lack of alternative pathways* to deal with problem: other services unable to cope with problem, or had transferred them to ED |
| Ahl et al  2006^[[1]](#footnote-1)^ Sweden | Ambulance | To analyse and describe patients’ experiences related to the decision to call an ambulance and when waiting for it to arrive.  *“The issue of inappropriate use of ambulance transport has mainly been studied from the caregivers’ perspective. To further understand whether or not patients use* *ambulance care in an inappropriate manner, and why, it is important to obtain an overall picture of the patients’ existential situation at the time they call an ambulance.” (p12)* | 2 | Exploratory interviews;  20 adult patients | *1. Making up one’s mind:* major decision, others often involved  - Situation experienced as intolerable, must get immediate help; someone else points out urgency of need; - Realising that other options have been exhausted after trying to manage on their own; overcoming hesitancy;  - Ambulance fastest, most safe and secure form of transport, enabling immediate access to care;  - Emphasising the need for care, including to others. *2.* *Waiting for help*:  - Experiencing the wait as long; desire immediate help; - Alone with one’s feelings - anxious, afraid, neglected, lonely;  - Avoiding/handing over responsibility – feeling of relief; sense of safety & trust |
| Becker et al  1993 USA | Mixed emergency/ urgent care: range of services unclear, mostly ED | To study the significant variables related to individuals’ response to asthma. *“… an ethos (…) surrounds ED use by asthma patients...” “Eighty-five percent of all visits to EDs have been found to be for non-life-threatening reasons…” “staff may regard persons whose lives do not appear to be in danger as wasting their time.” (p306) “Health professionals may assume that persons with asthma who frequent emergency services are not taking proper preventive measures, and are therefore at fault for their asthma being out of control.” (p307*) | 2 | Monthly semi-structured interviews (x3) & symptom diary; 95 adult patients | *Confrontation with health care: “Two major themes related to control shaped individuals’ experience of their asthma: self-reliance and self-mastery.” (p308)* - Unpredictability of the condition – affected efforts to control, created uncertainty; nervous, threatened or irritated; memory of previous severe/ unexpected episodes shaped future responses  - Discovering the limits of control; learning to identify markers of danger; extending boundaries of control through medication use; trying to avoid use of healthcare system  - The decision to seek medical treatment; previous experiences of being criticised for attending too early or delaying use of services; identifying a point when help needed; juggling assessment of the condition with other responsibilities, desire for self-reliance, and fear of death; questioning knowledge of professionals and effectiveness of treatment  *Dilemma of seeking urgent care:*  - Narrow definitions in healthcare 🡪 balancing delaying too long with seeking help too soon, fear of judgement  - Cultural assumption of personal responsibility for illness 🡪 stigma and blame for unpredictable episodes; but services fail to cure or manage illness effectively |
| Berry et al 2008 USA | PED | To identify parents’ reasons for choosing the ED over their primary care provider (PCP) for non-urgent paediatric care | 1 | Semi-structured ethnographic interviews; 31 families (37 parents) of children (age limit not specified) | *- Problems with PCP:* long wait for appointment; frustration with negative attitudes of staff at PCP office; communication problems with staff re how to get appointments; unhelpful, confusing explanations, strong accents  - *Referral by the PCP:* told to come to ED by staff or other services  - *Advantages of the ED*: efficient, faster service; ED resources including tests and X-ray; convenience of walk-in aspect; quality of care and confidence in care – more thorough; PED expertise with children, more child-friendly  - *Parent education*: most PCPs had not provided any info on signs to look for to know when to go to ED, urgent vs non-urgent conditions; some had discussed |
| Booker et al  2013 UK | Ambulance | To explore and understand patient and carer decision making around calling an ambulance for primary care-appropriate health problems | 1 | Semi-structured interviews; 16 adult patients or carers of adults | *Main theme: Patient and carer anxiety in urgent care decision-making*  Sub-themes:  *- Perceptions of ambulance-based urgent care*: perceived as competent to deal with anything; looking to service for rapid assessment, decision-making and signposting;  - *Perceptions of community-based care:* can’t provide the help needed, especially by phone; OOH service seen as limited;  - *Influences of previous urgent care experiences in decision making; interpersonal factors*: prior negative experiences with other services – wasting time, being referred on, unable to access GP, transport difficulties;  - *Patient and carer anxiety and decision-making:* need for urgent reassurance, sometimes as a result of health professionals’ advice on phone;  - *Interpersonal factors and the assessment of risk in decision-making:* others often involved, particularly carers – less likely to take risks, may drive person to do what carer thinks |
| Brousseauet al  2011 USA | PED | To better understand parental decisions to seek care for their children and physician perceptions of parents’ decisions to seek non-urgent ED care | 1 | In-depth interviews;  26 parents of children [& 20 PCPs] of children (age limit not specified) | - The need for immediate reassurance that their children are safe from harm is critical to parents’ decisions: looking for answers that satisfied worry; phone diagnosis not as reliable; ED seen as superior, some dissatisfied with PCP; reassurance more important than cost;  - PCP offices lack specific tests and treatments that parents & physicians believe may be necessary regardless of whether they are actually needed: ED seen as equipped to handle everything;  - Discrepancies exist between PCP and parent perceptions of adequate communication and access: parents did not think they were given education re appropriate ED use, although PCPs considered they did; parents did not feel they were seen as quickly as they wanted, although PCPs felt they were accessible;  - Non-urgent ED visits are not perceived as a significant enough breach in continuity of care to warrant significant concern. |
| Calnan 1983  UK | ED | A comparison of the processes involved in the decision to seek medical care for sufferers with different types of complaint. *“Explanations of patient use of the hospital accident and emergency department have, until recently, been coloured by provider’s conceptions of how the service ought to be used. Emphasis has been placed on examining why patients did not go to their GP.” (P151)* | 1 | Semi-structured interviews;  575 adult patients | - *Illness behaviour of patients with ‘minor’ cuts:* decision made quickly, usually straightforward – visible problem, sometimes familiar, known cause, mostly accidental; influenced by depth of cut, blood loss, part of body, child involved, need for particular treatment e.g. tetanus, stitching; influence of others e.g. authority figures, social network, sense of responsibility for individual; balancing family commitments with personal needs; decision to use ED not PCP more likely to be made if outside home or made by others; lack of GP availability and facilities to treat;  - *Illness behaviour of patients with minor illness:* more complex decision – often didn’t know what was wrong due to unfamiliar/unexplained/persistent symptoms; previous health history sometimes gave insight; more likely to have tried to contact GP than those with cuts, but often referred to ED by PCP staff, couldn’t wait for appointment, not satisfied with treatment or had no access to GP; other people excluding close family less influential in decision-making. |
| Capp et al 2016  USA | ED | To clarify from the patient’s perspective why adult Medicaid enrollees who want to receive care co-ordination services to improve primary care utilisation frequently use the ED | 2 | Secondary analysis of 1-2 page interview summaries from RCT;  100 adult patients | - Negative personal experiences with the health care system, especially PCP: lack of continuity, not listened to, treated differently because had state insurance – negative comments, made to feel a nuisance;  - Challenges associated with having low socioeconomic status: managing complex living situation a priority over health concerns; hard to remember appointments; transportation a barrier to PCP, some lived close to ED or called ambulance;  - Significant mental and physical chronic disease burden: most had chronic illness; no preventative care, responded when problem or exacerbation arose, then unable to access PCP quickly enough; past and recent traumatic life events leading to anxiety/depression. |
| Chin et al  2006 USA | PED | To understand patterns of decision making among families presenting to a PED for non-acute care and to understand pediatric ED staff responses | 1 | In-depth semi-structured interviews; 12 family caregivers of child (age limit not specified) [and 19 ED and P-ED staff members] | - *Patients were referred to the PED for non-urgent care by PCP office:* not their choice, mostly puzzled and unhappy;  - Outlier case – one person opportunistically capturing an episodic acute event, anxiety heightened by own past health experience  - *Complexities of poverty and competing priorities:* managing multiple responsibilities with few resources – ED referral added to problem; own needs did not match system priorities; primary care system complex and inflexible, easier to use ED;  - *Mistrust:* seen in previous study in relation to PCP, but not major theme here; some reservations re lack of understanding of black/white differences in beliefs re child nutrition, lack of sensitivity to other issues. |
| de Bont et al  2015 Netherlands | GP OOH | To explore experiences of parents when having visited GP out-of-hours services with their febrile child | 2 | Semi-structured interviews;  20 parents of child under 12 | - *Cautiously seeking care:* additional symptoms or problems prompt contact; initially wait then definite decision to seek care; GP had no time, problem out of hours, greater worry at night when unable to monitor easily;  - *Discrepancy between rationality and emotion:* anxiety increases with temperature – seen as indictor of illness severity; emotions take over; duration of symptoms important; parents of older children less anxious;  - *Expecting reassurance from a professional:* that they were caring correctly; wanting to know cause, nothing serious; physical examination important and GP expertise, not expecting medication; different doctor provides second opinion, used to seeing different people;  - *A need for consistent, reliable information:* consulting others including internet before seeking help but not being reassured; would value written information from GP for managing on another occasion, but needs to be from reliable source. |
| Durand et al  2012 France | ED | To explore the reasons why people with non-urgent complaints choose to come to EDs, and how ED health professionals perceive the phenomenon of “non-urgency | 1 | Semi-structured interviews; 87 adult patients [and 34 ED staff] across 10 EDs | - *To fulfil health care needs:* alleviate pain or discomfort and anxiety generated by the complaint; pain an emergency; needing reassurance  - *Barriers to primary care providers:* difficulty obtaining appointment; only alternative to accommodate work schedules; discerning health consumers – knew the system, assessed alternatives and made choice;  - *Advantages of the ED:* availability of diagnostic tests and treatment; convenience, being cared for in a single place, availability of medication. |
| Fieldston et al  2012 USA | PED | To elicit and to describe guardians’ and health professionals’ opinions on reasons for non-urgent PED visits | 2 | Focus groups: 3 groups of guardians of child under 5 (n=25) - most had taken a child to ED, but no indication of appropriate-ness of visit  [and 3 groups of paediatric health professionals (n=42]] | - *Perceived medical need:* need timely reassurance about concerns, especially if worried about symptoms; particularly for newborns and first born children; anxiety increased by awareness of negative outcomes for other children; better treatment, faster, more tests and interventions, second opinion – willing to wait;  - *System design, accessibility, availability:* aware of PCP systems but preferred convenience of ED, own schedules didn’t fit with PCP system, can’t miss work; OOH PCP phone access helpful |
| Goeman et al 2004^[[2]](#footnote-2)^  Australia | ED | To explore the reasons why individuals recurrently present with asthma to hospital EDs.  *“Episodes of severe asthma should be mostly preventable with current best treatment, yet asthma remains one of the most common reasons why patients seek emergency care”. “A number of ideas have been proposed as to why some individuals recurrently seek emergency care for asthma. “Effective interventions to prevent ED re-attendance for asthma will depend on understanding why this occurs.” (p113)* | 2 | In-depth semi-structured interviews;  32 adult patients | - *Reasons for emergency department attendance*: respiratory tract infection, shortness of breath, concerns re medication use, cost of medication use, run down/weather, desensitisation;  - Most had chronic severe asthma and only a few of these attendances considered preventable with reduced medication cost or increased knowledge of asthma management;  - Of those with less severe asthma, some attendances a result of low knowledge, poor medication use, lack of access to specialist care, lack of medication review, medication cost;  - Quantitative information indicates that more re-attendees had severe chronic asthma, more admissions and more asthma attacks than non-re-attendees in comparison group |
| Goepp et al  2004  USA | Service use decisions, particularly PED | “Low acuity use of EDs is often viewed as misuse or abuse. We designed a program to help users access services more efficiently.” (p522) | 1 | Participatory action model and ethnographic study: observation, interviews, groups;  90 families [interviews and focus groups with workers] | Initially designed a program using lay community workers to educate families about health care system and measure change in use of PCP and PED.  Negative reaction from participants and recognition of their needs led to change to qualitative methods to understand factors driving people’s health-seeking behaviour in order to educate healthcare professionals. Findings of ethnographic evaluation:  - *Fear and suspicion of healthcare providers and system:* experiences of discrimination and humiliation; hospitals dangerous, may perform secret medical experiments;  - *Differing definitions of health*: standard biomedical measures e.g. immunisation didn’t match families’ perceived need where housing, food, employment and safety were priorities; ED preferred due to timing and access issues, shorter waiting times – decision re service not made in relation to acuity of illness;  - *Systems complexity:* demands of living with poverty a barrier to efficient use of healthcare, appointments missed due to other priorities; ED offered easy access anytime, no need to make appointment or be blamed for missing them; size of hospitals & clinics intimidating, feel lost and unwelcome;  - *Value of liaisons:* support workers helped break down barriers of trust and complexity, support to navigate systems |
| Guttman et al  2003  USA | ED & PED | To identify reasons for medically non-urgent ED visits from the users’ perspective…. to identify through their reasons what may have prompted them to use the ED for medical care they could have ostensibly received in primary care sites. | 1 | Structured interview protocol with open-ended questions and scaled items re decision; 331 parents/ guardians of child up to 18 and 77 adult patients | *1. Conceptions of needs:*  *- Relief from pain or discomfort:*  especially when a child; pain can be seen as an emergency;  - *Reassurance:* that condition not dangerous or at risk of deterioration, not at fault; especially for children;  - *Official approval:* to undertake particular activities, e.g. holiday;  - *Caretaker responsibility:* don’t take chances with child, even when situation doesn’t seem an emergency; seek help when symptoms persist, especially at weekends; parents also seek help for self so they can care for child;  - *Recourse, second opinion and referral*: especially when not satisfied with PCP consultation, treatment, advice; wanting referral to specialist;  - *Financial:* lack of insurance; no regular doctor, recently moved, out of town, nowhere else to go;  *2. Conceptions of Appropriateness:*  - *Worrisome condition:* anything causing concern is appropriate, especially for children;  - *After-hours office services:* ED appropriate when PCP unavailable; accommodates work schedules;  - *Perceived unavailability of timely appointments in primary care settings:* scheduling difficulties, long waits, can’t tolerate delay; no appointment needed for ED; especially for public clinic users;  *3. Preference:*  *- General preference:* ED close, familiar, trusted, used as primary care site;  - *Facilities and staff:* tests, medications; one-stop healthcare; better doctors; treated with more respect; some cultural and educational variation around these factors;  - *Shorter wait:* compared to PCP  Develop a typology which maps congruence between conceptualisation of emergency and user’s own reason against user’s preference for ED to identify 4 types of user:  “No alternative”/“Prefer the ED”; “Would rather go elsewhere”/  ”ED is the best option for an emergency”. |
| Haqiqi et al  2016  Canada | PED | To explore the reasons that lead parents to select the ED over a dental clinic for their child’s non-traumatic dental problem. | 1 | Semi-structured interviews;  15 parents of child under 10 | *1. Family-related barriers:*  - *Parents’ understanding of oral health:* (wait and see attitude, lay diagnosis);  - *Parents’ socioeconomic challenges:* balancing care for child with other demands including irregular/long hours, dental problems not priority;  2. *Provider-related barriers:*  - *Poor access to dental care:* limited for children; dentist referred families to the hospital due to complex/multiple problems or child’s behaviour (crying, fear), hospital the last resort; dentist unavailability - closed, long wait;  - *Poor quality of dental care*: perceived lack of patience with children, lack of competency;  *3. Satisfaction with care provided at the hospital* – will not consult private dentists again. |
| Hopton et al  1996  UK | GP OOH | To investigate patients' accounts of calling the doctor out of hours.  *“Alongside the debate about factors influencing demand for out of hours care is debate about the appropriateness of the demand. Substantial published work acknowledges that lay views of health problems and what should be done about them often differ from those of professionals, and the debate about appropriateness often hinges on this discrepancy. Despite this evidence and calls for patient education as a means of tackling increasing demand and inappropriate use, patients' perspectives on out of hours calls have been neglected.” (p991)* | 2 | Semi-structured interviews; 23 adult patients or calling on behalf of an adult and 23 parents of child under 16 | - *Symptoms:* ideas about normal and abnormal illness, including severity, unrelieved, unexplained, combination, sudden onset, long duration, high temperature in child, behaviour;  - *Context of call:* concerns about specific illness; caller’s feelings – panic, distress; responsibility for others – especially children; previous attempts to manage the problem including self-care, lay and professional advice, wait and see then taking action; failing to get an appointment; lack of medication in house;  - *Previous experiences of health services and health professionals:* past frights when things more serious than expected; current concerns about other illness; lack of confidence in health professionals, feeling not taken seriously; previous medical successes prompt quicker action now;  - *Outcome of calls:* mostly prescription, referred to hospital; result affected view of future actions – most would do the same again. |
| Houston & Pickering 2000 UK | GP OOH | To investigate how parents use the GP out-of-hours service.  *“The apparent increase in out-of-hours GP consultations is a source of considerable concern*  *and debate. The underlying premise of much of this concern is that many of these calls are unnecessary and the result of a combination of*  *over expectation and inadequacy on the part of those making the request for consultation.” (p234)* | 2 | In-depth semi-structured interviews; 29 families of child under 10 | - *Belief in self-management:* desire to cope and take responsibility for child;  - *Strategies for managing childhood illness:* range of approaches, including temperature, medication;  - *Responsibility and fear of making the wrong decisions:* wanting to do the right thing, feeling ill-equipped to manage, especially as new parent  *- A real dilemma:* whether or not to call doctor, aware of demands on service, but want to do right thing for child;  - *Calling the doctor:* call triggered by combination of emotional response and particular situation; linked to previous experiences of persistent calls leading to eventual help; loss of confidence in own strategies;  - *Social support:* affected whether needed to call doctor e.g. if no family near, single parent, social isolation at night;  - *Previous healthcare experiences:* those with more frequent prior contact more likely to call, more willing to hand over responsibility, disempowered by past experience especially if serious – less able to manage minor illness;  - *Differences between callers and non-callers:* non-callers emphasised managing and being seen to manage more; callers believed entitled to use service, best thing for their child. |
| Howard et al  2005  USA | ED | Why do people choose to come to the ED instead of their PCP with non-urgent medical complaints? | 1 | Interviews using open-ended interview tool modified from survey questionnaire; 31 adult patients (18-50) | - *People used the ED because they have been told to do so by staff in their PCP’s office:* positive re PCP but long wait for appointments; told to seek help at ED by office staff rather than health professionals;  - *People have difficulty gaining an appointment with a PCP in a timely manner:* perception or experience of being unable to get experience on the day they called; PCP not in or not open, leave messages and long wait for reply;  - *Time played a factor in every response given by the participants:* hard to get appointment, long wait in office then only seen for very short time; having to schedule work around appointment, needing to see doctor on the day if off sick so can get back to work quickly; childcare an issue, can’t sit in doctors for ages with kids. |
| Hugen-holtz et al  2008  Nether-lands | GP OOH | To gain insight into the health-seeking behaviour parents who ask for immediate medical attention for their children.  *“Data from GP co-operatives … show that children make more use of OOH care than members of other age groups, although the health problems … are less urgent from a medical point of view.” “Knowledge about underlying health beliefs is relevant if we want to close the gap between parents who ask for immediate medical attention for their children at all times, and doctors who feel that some parents are making inappropriate use of OOH facilities.” (p173)* | 2 | Semi-structured interviews:  27 parents of child up to 16 – 19 with minor illness; 8 requiring immediate referral to hospital | - *Knowledge of parents and their actions at home:* used existing knowledge of child’s behaviour and appearance to determine they were sick – deviation from norm first sign something wrong; used diagnostic procedures before contacting for help – fever a particular concern/trigger;  - *The turning point:* most had thought of medical diagnosis before calling; sought help when felt incapable to handle situation – symptoms worsening/alarming/inexplicable, child’s discomfort, own approach failed; intuition important; seeking contact became the only way to get relief from worry;  - *Not wanting to take a risk:* didn’t trust situation, own feelings a trigger to seek help; worry about leaving things too long in case something serious, don’t take a risk, go too often rather than not enough; seeking examination and reassurance rather than treatment, want to know what’s wrong, rule out serious illness; risk avoidance part of parental role – child important, have to watch closely, responsible for getting help on time, stand up for their children. |
| Hunter et al  2013  UK | Mixed emergency care: ED, OOH and walk-in centre | To elaborate on the processes by which patients with long-term conditions choose between available options for care in response to a health crisis, to inform the development of future policy and guidance on modifying emergency care use.  *“…health policy in many countries seeks to constrain and shape patients’ care decisions in order to ensure that the service accessed reflects the level of medical need. Specifically, policies seek to reduce use of hospital emergency department care, mainly because of its high cost compared to alternative healthcare options.” (p335)* | 2 | Semi-structured interviews:  50 adult patients | *1.* *Patients framed instances of emergency care as unavoidable:* reluctant to use, don’t want to be a burden, no other option due to seriousness of problem;  2. *Previous experiences shape future emergency care use:*  - *Negotiating and establishing urgency:* base future decisions on previous experiences and responses of social network and health professionals, decision sanctioned or made by someone else;  - *Prioritising technological expertise over established relationships in times of crisis:* emergency care services have facilities not available in primary care; disease-specific care also better in specialist clinics; WIC/OOH provision don’t provide benefits of ED or of GP, so not preferred;  - *Judging accessibility of services:* range of barriers to unscheduled access to primary care, mostly organisational – appointment systems, transport/travel, long waits. |
| Keizer-Beache & Guell  2015  St Vincent and the Grena-dines, Caribbean | ED | To explore attitudes of non-urgent accident and emergency department patients in a middle income healthcare setting … to understand how and why they decide to seek emergency care and resist using primary care facilities. | 1 | Semi-structured interviews;  12 adult patients | - *Habitual use of the ED:* a default process rather than deliberate decision, seen as general societal behaviour; encouraged by family, friends, colleagues;  - *Systemic encouragement of the use of the ED:* limited scheduling and hours of primary care clinics reinforced used of ED, also lack of diagnostic facilities; referred to ED by clinic staff;  - *Deliberate use of the ED:* some making active choice to use; transport, convenience; seriousness of complaint; positive previous experience – quality of care, time available. |
| Koziol-McLain et al  2000  USA | ED | To gain an understanding of the context in which patients choose to seek health care in an ED.  *“The policy goal of shifting non-urgent visits from the ED to nonemergency health care settings is commonly devised, planned and implemented without considering patients’ perspectives.” “Despite an increase in acuity among ED patients in past years, the majority of patients do not come to the ED because of life-threatening problems.” (p554)* | 1 | Unstructured interviews;  30 adults | - *Toughing it out:* putting up with things before going to ED  - *Symptoms overwhelming self-care measures:* mostly use of over the counter medicines; decision made when problem began to impact on function;  - *Calling a friend:* seeking support and advice from friends and relatives, especially mothers;  - *Nowhere else to go:* couldn’t access non-emergency care; being referred to ED by other healthcare providers;  - *Convenience:* work schedules, child care and transportation barriers affecting choice of ED. |
| Lawson et al 2013  USA | ED | To explore the reasons for asthma-related ED use among adults.  *“ED visits for asthma have long been considered “avoidable” yet exacerbations remain common.” (p1) “…it has never been more important for health systems to find new ways to reduce the number of ED visits and hospitalisations for asthma.” (p2)* | 2 | Semi-structured interviews;  26 adults | *1. Reasons for ED utilization:*  - *ED as a fast or convenient site of care:* can’t wait for clinic appointment  *- ED resources or expertise:* know what to do, treat the condition frequently  - *Inability to access outpatient provider:* symptoms worse at night  - *Inability to access medication:* not picking up medication due to work  *- Lack of symptom improvement:* tried medication but not helping  - *Severity of symptoms:* know that it’s bad enough to need ED  - *Referred by outpatient provider*  - *Told to go to ED by friend or family member*  *- Insurance status:* ED will see if have no insurance  *2. Definitions of flare severity* – symptoms grouped into mild (can manage) or severe (can’t manage themselves); lack of recognition of middle ground where should try to escalate medication and seek outpatient help. |
| MacKichin et al  2017  UK | ED | To describe how processes of primary care access influence decisions to seek help at the ED.  *“Given that a significant proportion of ED attendances.. are discharged with ‘advice only’… and that ED attendances peak during the working day on Monday, it is presumed that better access to primary care will relieve pressure on EDs” (p3)* | 1 | Ethnographic study of 6 GP practices: observation of reception areas, document analysis, interviews;  20 patients & 9 parent/ carers  [19 clinical and non-clinical staff] | - *Intricate appointment systems:* difficult to understand, different in each practice, frequently changing; receptionists seen as gatekeepers  - *Appointment availability:* increased use of triage, telephone and same day appointments; less routine slots; confusing range of appointments;  - *Communication and talking on the telephone:* language and other barriers sometimes driving ED use  *- Is it an emergency?* Differing understanding between staff and patients re definition of urgent, cultural issues can affect;  *- Out of hours care*: lack of knowledge and understanding re how to access; perceived/experienced as poor quality;  *- Perceptions about level of care accessible at GP practice*: ED seen as quicker way to access care; higher level of skill, more specialist and better quality of care; parents’ risk perception - viewed child’s problem as urgent so ED seen as more appropriate, better. |
| McGuigan & Watson  2010  UK | ED | To discover the factors influencing patient decisions to attend EDs for non-urgent treatment | 1 | Short semi-structured telephone interviews (mean length 3 minutes);  196 adults | Reasons for attendance:  - Regarded their conditions as serious and in need of urgent attention  - Could not obtain appointments with GPs  - Had been advised to do so by, for example, families, friends, pharmacists, healthcare helpline advisers or GP receptionists  - Expected their GPs to send them to EDs anyway  - Knew nowhere else to go for help  - Were not registered with GPs  - Lived near to an ED  - Type of illness/injury (soft tissue injuries largest group) and duration of symptoms (lasting longer than expected)  - Diagnostics (especially X-rays) – expectation of need for tests only available at the ED |
| Neill et al  2014  UK | Service use decisions | To examine how parents of children under five years, from a range of socioeconomic groups… use information to make decisions during acute childhood illness at home.  *“Consultation rates for children are rising, yet little is known about factors that influence parents’ help-seeking behaviours…. Professional and political solutions have not reduced demand; therefore, collaborative approaches …are now needed to improve parents’ access to information” (p1) “Ongoing epidemiological research aims to identify those most likely to consult, so interventions can be targeted at these groups. This must be balanced with concerns about discouraging service use by children who need urgent care.” (p2)* | 2 | Focus groups and interviews;  27 parents from South Asian, Travelling and White British communi-ties | - *Effect of the nature of the child’s illness on help seeking:* child’s distress, symptom duration or unfamiliarity important, fear of serious illness;  - *Experience and knowledge and their influence on help seeking:* instinct/ experience important; more concern in first-time parents; previous failure to recognise serious illness can undermine confidence; some just worry more;  - *Social support and its impact on help seeking behaviours: value of social networks* – Travelling/S Asian most likely ask parents, White British ask family who are HCPs or lay experts; other resources used to check legitimacy of using health services; single parents’ logistical problems accessing help; timing of day/week affects decision  - *Access to health services:* difficulty getting GP appointments leads to use of other services; NHS Direct used to check legitimacy of help-seeking; talking to receptionists a barrier, especially with language; WICs not valued – no continuity, unwilling to prescribe, refer back to GP;  - *Trust in service provider and effect on help-seeking behaviour:* affected by existing relationship and experiences; loss of trust when illness missed, lack of examination, ineffective treatment, conflicting into, not answering questions, referring on;  - *Social expectations and influence on parents’ help seeking behaviour:* wanting to do the right thing for child and in the eyes of healthcare professionals and society – feeling inferior to doctors, being labelled as inappropriate user, worry about being criticised, not taken seriously in future. |
| Olsson & Hansagi  2001  Sweden | ED | To explore what lies behind repeated emergency department (ED) use, from the patients’ own perspectives.  *“A subgroup of patients make frequent use of hospital EDs, thereby accounting for a substantial portion of the total number of visits to these facilities.” “Repeated visits may frustrate the staff… as these patients’ complaints are often judged as non-urgent and inappropriate for ED care” (p430)* | 2 | In-depth interviews;  10 adult patients | Key points:  - *Symptoms are perceived as a threat to life and to autonomy*: fear of dying; previous trauma – own or in others – triggering increased concern; trying to maintain autonomy but feeling of powerlessness forcing help-seeking;  - *Struggles with adverse life circumstances:* scarce resources, health problems, poor support, precarious situations;  - *Needing frequent help associated with feelings of inferiority:* wanting help-seeking to be respected, satisfaction with care reduced when feel their use is classified as inappropriate or when symptoms are belittled  - Occasional referrals to psychiatrist do not seem to lead to any continuous treatment or change in help-seeking behaviour |
| Shaw et al 2013  USA | ED | Exploring the decision-making processes to use the ED for non-urgent needs of medically underserved patients. | 1 | Semi-structured interviews;  30 adult patients | *Two subgroups: with and without knowledge of alternatives:*  *1.* *No knowledge of alternative primary care options*: no PCP; belief that only ED available if had no insurance;  *2. Knowledge of alternatives:*  - *instructed by a medical professional:* specialists and PCP;  *- access barriers to regular source of care*: PCP closed, no appointment in time, long wait at clinic, negative past experiences;  - *perceived racial issues with the FQHC:* discomfort as no other patients from own racial background; perceived to provide care for immigrants which is assumed to be poorer quality;  - *defining healthcare need as an emergency requiring immediate attention:* different places for different needs, ED most appropriate in this situation;  - *transportation/location:* most had no car, proximity to service important;  - *cost of care:* if no insurance PCP require payment upfront, ED bill afterwards; balancing ED costs with future costs to own wellbeing. |
| Stafford et al  2013  UK | Mixed emergency/urgent care: ED, walk-in centre GP OOH service | To explore why patients with simple mechanical back pain seek urgent care. | 1 | Semi-structured interviews;  11 adult patients | - *GP access:* unsuccessful in getting an appointment; directed to urgent care; surgery closed;  - *Pain and analgesia:* pain intensity, desire for quick relief;  - *Impaired function:* walking, daily living, child care – distress motivating help-seeking  - *Different:* unlike previous episodes; prompted fear, frustration, anger and pessimism  - *Concern that something wrong;*  - *Investigation:* wanting further examination to prove there is a problem or to understand problem;  - *Third party:* advised to use urgent care by healthcare professionals or family; use this route directly on subsequent occasions;  - Repeat visits: continue to go back even though not satisfied with outcomes. |
| Wilkin et al  2012  USA | ED | What factors influence residents’ [in a low income urban community] decisions to use emergency versus primary care? | 2 | Community forum discussions;  3 groups of 12-21 adults from area with high 911 use (only some participants identified as 911 users) | - *Knowledge related to using emergency versus primary health care options:* understanding the concept of emergency but hard to be distinguish in a real situation;  - *Available health care services:* knowledgeable about local services, but didn’t think they could meet their needs due to lack of specialists;  - *Attitudes about emergency and primary health care:* frustration with PCP referrals out of area; long wait times for appointments and to be seen (also at ED); poor customer service in clinics – public discussion of personal matters, rudeness, judgement;  - *Barriers to primary care:* affordability of health care especially if lack of insurance; complex paperwork for reduced price health care; transportation difficulties leading to missed PCP appointments, no awareness of free transport services. |
| Woolfen-den et al  2000  Australia | PED | Explored the parental attitudes, perceptions and beliefs that play a role in the use of a tertiary PED when a child has a non-urgent illness. | 1 | In-depth semi-structured interviews;  25 parents of children (age range not specified, sample includes up to 14) | - *Parental triage:* factors influencing interpretation of severity – fever, breathing, pain, vomiting, change of symptoms, lack of resolution with treatment, age, first child, medical history;  - *Expertise*: PED seen as having greater knowledge, training, experience, child-specific; setting more child-friendly; feeling helpless, guilty, stressed; responsibility to relieve suffering; greater confidence as children older;  - *Access:* lack of acceptable local healthcare, especially out of hours; own GP unavailable; prefer PED even when local services accessible;  - *Parental expectations:* dissatisfied with local services – poor communication, feeling rushed, not treated as individuals, inconsistent or unclear explanation and advice; seeking reassurance from expert. |

**QUANTITATIVE STUDIES FOR TESTING THE PROGRAMME THEORIES**

| **Author, Year and Country** | **Service** | **Stated aim/objective, *plus additional text where this provides justification for inclusion of the paper*** | **Relevance** | **Data collection method; number of participants** | **Key themes/issues identified** |
| --- | --- | --- | --- | --- | --- |
| Allen & Cummings  2016  USA | ED | To identify differences in non-urgent and urgent Ed use between Hispanic and non-Hispanic people with different levels of acculturation | 1 | Cross sectional population survey | White and acculturated Hispanic were more likely to make non-urgent visits |
| Atenstaedt et al  2015  UK | ED | To explore why non-urgent patients attend an ED despite alternative services | 1 | Survey of convenience sample of 806 ED attenders | Half sought advice from others beforehand: friends (31%) or GP (26%)  Some had tried to use GP, Minor Injury Unit, NHS Direct, GP OOH, pharmacy  Those who didn’t try an alternative first thought that ED was the best place because they thought they might need an x-ray (46%), believed the GP was unable to help (29%), wanted a second opinion (3%), wanted a specialist/hospital (19%), found ED easier or nearer to get to (8%), believed it would be quicker at ED (11%), or said the GP was not available (19%)  3% were not aware of other services  20% would have changed their visit to ED if they had known about alternatives |
| Benahmed et al  2012  Belgium | PED | To identify factors associated with non-urgent use of EDs for paediatric patients | 1 | Survey of 3117 children aged <15 years comparing appropriate and inappropriate attendance | 40% identified as inappropriate in that no tests undertaken and walked in  Inappropriate attenders were more likely to be: Aged under 2, have a GP, live closer to the ED, attend out of hours, and come from disadvantaged families (but not after adjustment)  No difference for chronic patients, gender |
| Carret et al  2009  Brazil (first author) | ED | To identify the factors associated with inappropriate use of EDs | 1 | Systematic review of 22 studies, n=most of which were cross sectional but allowed comparison between inappropriate and appropriate use | 20%-40% of patients identified as inappropriate in studies  More likely to be younger, females, have no comorbidities, had to pay for GP, have no GP, difficulty in accessing primary care (getting appointment, not open, need to wait)  Duration of symptoms associated with inappropriate attendance (direction of effect is not stated)  Day of week, distance from ED, and marital status/living alone were not associated with it  There were mixed results for socioeconomic status, working full or part time, ease of getting tests and treatments, better care, dissatisfaction with GP, means of transport |
| Cheek et al  2016  Australia | ED | To identify factors associated with low-acuity use of ED amongst GP and self-referrals | 1 | Cross- sectional survey of 138 low-acuity patients | ED had expertise they needed was most common reason. All tests and seeing doctor in the one place.  Not happy with time needed to wait for GP (20% of 45 in-hours presentation had not been able to get a GP appointment)  ED closer to work or home than GP  ED does not charge for tests  27% referred by GP, some because GP had no appointment, GP wanted second opinion or GP felt they did not have the equipment needed  31% thought GP would send them there anyway  Solution the patients wanted was more GP OOH with x-ray facilities that they did not have to register for |
| Deavenport-Saman et al  2016  USA | PED | Explore why children with autism use EDs compared with those without autism, because they are known for over utilising EDs for non-urgent visits | 1 | Routine data from ED on 115,443 children aged 2-21 years | Autistic kids more likely to have non-urgent visits  Used Andersen’s Model of Health Service Use to show that older children, non-Hispanic, and children with private or no insurance were less likely to have non-urgent visits. People were more likely to attend for non-urgent visits when other services were open |
| Diserens et al  2015  Switzerland | ED | To test if the characteristics of adult patients attending Ed with non-life threatening conditions changed over time between 2000 and 2013 | 1 | Survey of 516 attendees in 2000 versus 581 in 2013 | Increase in self-referrals (52% vs. 69%), delays of more than one week, belief did not need hospitalisation  Decrease in trauma (34% vs. 24%), and consultations within 12 hours of onset of symptoms (54% vs. 31%)  Most common motives for attending Ed were unawareness of alternatives, dissatisfaction with GP treatment or appointment.  Two urgent care centres had opened in the area in between the two time periods. Concludes that ED used as convenient primary care |
| Durant & Fahimi  2012  USA | Ambulance | To understand the characteristics of patients who use ambulances to attend EDs for low-acuity conditions | 1 | Routine data comparing those arriving at ED by ambulance with those arriving in other ways | Hypothesised that more vulnerable groups would be non-urgent  Looked at non-urgent users in ED and compared who arrived by ambulance and who walked in  Ambulance classed as non-urgent at ED more likely to be: Older age, Medicare insurance,  Homelessness, attend OOH, have mental health problem or poisoning  No differences: day of week, region of United States, Hispanic or not, race, gender |
| Farion et al  2015  Canada | PED | To understand low-acuity visits to PEDs | 1 | Prospective cohort study using survey of 2443 tertiary PED parents of children <18 | 27% occurred within 4 hours of onset, and 5%-11% after four weeks from onset depending on whether they had a GP  Reasons included fever, ear ache, cough/respiratory, vomiting, rash, injury  25% sought care from GP, 21% from helpline and 19% from urgent care clinic  Parents attended PED because it had access to everything needed, offered care from paediatric experts, was trusted.  Patients without a GP had longer symptom duration and did not know of other services. |
| Faulkner & Law  2015  Australia | ED | To explore unnecessary use of EDs by older people | 1 | Routine data, focus groups with staff and interviews with 58 older people | Routine data reported as very limiting in terms of variables that can be tested  Patient interviews reported like a survey so not included in our qualitative review  Reasons given in patient interviews included: don’t want to wait for GP – even if 12 hours or 2 days; other person took them; had received good care before and expected to receive it again; exacerbation of a chronic condition so learned that ED was best and GP a waste of time; x-ray gets done at once with everything else so very convenient; need to speed. |
| Freed et al  2016  Australia | PED | Why parents of children aged 0-4 attend ED with lower urgency conditions | 1 | Survey of 1150 parents of children 0-4 triaged to lower categories | 43% attempted to make an appointment with a GP  2/3 of those contacting a GP were told to go to ED  Few attempted to use a GP OOH service (7%) or telephone service (20%)  Reason for attendance was: Seriousness of condition (94%), duration of illness (75%), injured kids need to go to ED not GP (68%) |
| Hjalte et al  2007  Sweden | Ambulance | To describe the characteristics of patients transported by ambulance but not needing this care, and comparison with those needing this care | 1 | Questionnaire and medical records. Comparison of 604 clinically unnecessary with 1373 necessary | Less likely: <16 years  More likely: abdominal and urinary problems  No differences: time of day, gender |
| Hodgins & Wuest  2007  Canada | ED | To explore reasons for attending ED with less urgent health problems using Andersen’s Model of Health Services Use | 1 | Structured interviews with 1612 patients triaged to the waiting room | Geographic location important – urban and rural areas differ  Importance of pre-determined items: Severity of symptom, getting worse, no other option, availability of GP, convenience, tests only available at ED, advice from family and friends  Some people had undertaken self-care measures before going to the ED: 68% used over-the-counter remedies, 10% -15% had seen a GP.  Those who were afraid were less likely to self-treat  Less willing to wait 2 days to see GP if in pain (disturbing symptom) in a rural setting, or had post school education |
| Kallestrup & Bro  2003  Denmark | GP OOH | To understand why parents of febrile children use GP OOH | 2 | Structured interviews with 146 parents with children under 12 | Fever, coughing, and ear pain were common problems  52% discussed the decision with others, 21% used over the counter drugs beforehand, 46% did not consider it serious, 49% felt helpless, 17% feared it was serious, 34% wanted symptom relief, 39% had it 3 days or more, 13% expected a prescription |
| Kawakami et al  2007  Japan | Ambulance | To investigate the influence of socioeconomic factors on medically unnecessary ambulance calls | 1 | Population survey of 2029 using hypothetical vignettes | More likely: male, elderly, no car, living alone  No difference: price, knowledge of other services, history of ambulance use |
| Keizer et al  2015  The Netherlands | GP OOH | To determine whether GP OOH visits for non-urgent problems are a result of patient beliefs or deficiencies in the healthcare system | 1 | Survey of 646 attendees, comparing medically unnecessary with medically necessary | 30% medically necessary compared with MU  Medically unnecessary attendees were: younger, frequent attenders, longer existing problems, belief that GP OOH was there for everything  Medically unnecessary attendees were worried (45%) and wanted information. Only 8% had poor access to GP or 6% no availability. |
| Kirkby & Roberts  2012  UK | Ambulance | To determine the characteristics of those most likely to call an ambulance inappropriately | 1 | Online convenience population survey of 150 using 5 hypothetical vignettes | No participant characteristics were predictive of calling an ambulance inappropriately once confounders were taken into account. Those with first aid training less likely to be inappropriate decision makers but not after confounding considered.  Highly convenient sample |
| Marks et al  2002  UK | Ambulance | To describe the characteristics of people not transported to hospital | 1 | Routine data on 498 people not transported | 34% were falls  Mainly elderly |
| Moll van Charante et al  2008  The Netherlands | GP OOH+ED | To determine the motives of those who self-refer to EDs compared with those contacting a GP OOH service | 1 | Postal survey of 224 self-referrals at ED and >5000 routine data for GP OOH | Self -referrals to EDs were predominantly young males with an injury. They had a strong preference for the ED and wanted the diagnostic facilities at the ED and the specialist service there. 26% said family played a role in the decision to attend  Patients with an injury were 3.6 times more likely to use ED than GP OOH  Parents of children aged 0-4 and older people aged >65 were more likely to use GP OOH  More likely to go to the ED if lived longer way from GP OOH  No association with social deprivation and setting |
| Patterson et al 2006  USA | Ambulance | To estimate the prevalence of medically unnecessary transports for children | 1 | Linked routine data on 5693 children aged 0-17 years, comparing medically unnecessary visits to ED with necessary | More likely to be non-white, rural, insured by Medicaid, young children <4, have behavioural problems  No difference for day of week |
| Penson et al  2012  UK | ED | To estimate the potential for patients with minor or moderate conditions to be managed in settings other than the ED | 1 | Survey of 261 ED attendees and notes review | Belief that only an ED could deal with it – fracture, need x-ray (approx. 50%)  30%-50% did not of GP OOH, walk-in centre or NHS Direct was available  30% not the first contact with a health service for that health problem  Advised by someone else – health professional more likely than family/friend  10% looking for second opinion  <7% for convenience or being seen quicker |
| Rajpur et al  2000  UK | GP OOH+ED | To compare reasons for using GP OOH and ED for primary care reasons | 1 | Structured interviews with 102 attenders | ED attenders more likely to be unemployed and white  46% of ED attenders had not contacted their GP before attending |
| Smith et al  2015  Canada | PED | To explore factors affecting use of PED for non-emergency concerns | 1 | Cross-sectional survey in tertiary PED, 300 parents | 32% attempted to contact another service first  59% instructed to attend the PED.  The top 3 reasons for attending: it specializes in children, familiarity with hospital, closest location to patient |
| Thornton et al  2014  New Zealand | ED | To identify reasons for attend an ED rather a GP for self-referrals to ED | 1 | Survey of 421 attenders | 23% who had a GP had contacted them prior to ED and 73% advised to go to ED  30 were admitted to hospital  14%-29% reported their GP was closed, depending on time of day |
| Uscher-Pines et al  2013  USA (first author) | ED | To identify factors affecting ED non-urgent visits | 1 | Systematic review of USA research, 26 studies that compared urgent and non-urgent as well as cross-sectional | % Non-urgent varied between 8% and 62% due to different definitions in use  More likely: younger adults (6/9 studies), convenience of ED compared with alternatives (3/3), poor access to GP (4/4), referral by doctor (1/1)  Mixed results – race (4/9 studies said Black people more likely), gender (4/10 said women more likely but 2/10 said men), income (2/4 said low income more likely), insurance (2/13 studies said uninsured more likely and 2/13 less likely), people with poor health (2/4 studies more likely), previous use of health care  Studies did not measure cultural norms or personality traits such as coping mechanisms, perceived severity of illness  >80% of non-urgent patients perceived that problem was severe |
| Walsh  1995  UK | ED | To identify the reasons why people attend EDs using the Health Belief Model | 1 | Structured interviews with 233 people with minor injuries attending an ED | ED better than GP (17%)  GP would send me here anyway (15%)  Quicker than GP appointment (15%)  Went to GP and sent here (12%)  Advised by others (12%)  More convenient that GP (10%)  GP not available (9%)  Conclusion that it is rational decision making |
| Watson et al  2015  UK | ED+GP+ pharmacies | To compare outcomes of consultations suggestive of minor ailments in EDs, GPs and pharmacies | 1 | Survey of 377 attendees at baseline and after the visit, comparing ED, GP and pharmacy | % symptom resolution was the same in all three settings  Pharmacy cost £29, GP £82 and ED £147  ED use was associated with perceived seriousness and short duration of symptoms  Convenience of location dictated choice of setting  ED users more likely to have musculoskeletal pain, employed full time, be single, perceive the problem to be somewhat serious, needed to be seen within a day, and if they had not experienced the symptom before  GP users more likely to be employed fulltime, perceive the problem to be somewhat serious, and to have had the problem for a long time |
| Williams et al  2009  Australia | PED | To understand why parents of young children attend a tertiary PED with non-urgent conditions | 1 | Survey of 355 parents of children. Mean age of 5.5, 45% over 5 years old | 68% rated the condition as moderate to very serious  66% sought advice before going to ED (GP, and advised to go to PED)  41% of non-injuries arrived 2-7 days after onset |
| Zhou et al  2015  UK | GP OOH | To test if difficulties in accessing a GP in-hours affects GP OOH use | 2 | National survey of GP use, 567 049 respondents | Worse in hours access was associated with higher use of GP OOH, particularly convenience of opening hours  The relationship was strongest for patients not in employment or education  Could reduce GP Ooh demand by 11% by improving access |

1. 2012 Ahl paper provided additional information relating to the same study, particularly methods and sample [↑](#footnote-ref-1)
2. 2002 Goeman paper provided additional information relating to the same study, particularly methods and sample [↑](#footnote-ref-2)
